# Classification of *Camellia* (Theaceae) Species Using Leaf Architecture Variations and Pattern Recognition Techniques

**DOI:** 10.1371/journal.pone.0029704

**Published:** 2012-01-03

**Authors:** Hongfei Lu, Wu Jiang, M. Ghiassi, Sean Lee, Mantri Nitin

**Affiliations:** 1 College of Chemistry and Life Science, Zhejiang Normal University, Jinhua, China; 2 Santa Clara University, Santa Clara, California, United States of America; 3 School of Applied Sciences, Health Innovations Research Institute, RMIT University, Melbourne, Victoria, Australia; American Museum of Natural History, United States of America

## Abstract

Leaf characters have been successfully utilized to classify *Camellia* (Theaceae) species; however, leaf characters combined with supervised pattern recognition techniques have not been previously explored. We present results of using leaf morphological and venation characters of 93 species from five sections of genus *Camellia* to assess the effectiveness of several supervised pattern recognition techniques for classifications and compare their accuracy. Clustering approach, Learning Vector Quantization neural network (LVQ-ANN), Dynamic Architecture for Artificial Neural Networks (DAN2), and C-support vector machines (SVM) are used to discriminate 93 species from five sections of genus *Camellia* (11 in sect. *Furfuracea*, 16 in sect. *Paracamellia*, 12 in sect. *Tuberculata*, 34 in sect. *Camellia*, and 20 in sect. *Theopsis*). DAN2 and SVM show excellent classification results for genus *Camellia* with DAN2's accuracy of 97.92% and 91.11% for training and testing data sets respectively. The RBF-SVM results of 97.92% and 97.78% for training and testing offer the best classification accuracy. A hierarchical dendrogram based on leaf architecture data has confirmed the morphological classification of the five sections as previously proposed. The overall results suggest that leaf architecture-based data analysis using supervised pattern recognition techniques, especially DAN2 and SVM discrimination methods, is excellent for identification of *Camellia* species.

## Introduction

*Camellia* is a large genus of family Theaceae with many species of significant economic and scientific value [1]. Some *Camellia* species are used to produce green tea, a popular beverage. It is estimated that more than 3.6 million tons of tea leaves are produced annually in 40 countries [2], [3], [4]. *Camellia* species offer a range of health benefits [5]. Some species are primarily cultivated as ornamental plants while the seeds of others are used as edible oils [6], [7]. This wide usage of the *Camellia* species has resulted in extensive cultivation and production. In China alone, more than 3 million hectares of agricultural land is used to grow *Camellia* species to produce in excess of 164,000 tons of edible cooking oil [5].

Although *Camellia* is grown in many regions of the world, it is particularly prevalent in East and Southeast Asia and its identification and classification has been the subject of many studies [6], [7], [8], [9]. Traditionally, professionals dealing with the production, distribution and sales of *Camellia* use their experience and intuition to classify the plants into categories with distinct economic values. Later, researchers developed different taxonomic and analytical methods for classification. In 1958, Sealy [8] reported 82 *Camellia* species that he classified into 12 sections. More recently, Chang [10] grouped the native Chinese *Camellia* into four subgenera, 22 sections, and 280 species, whilst Ming [6] arranged them into two subgenera, 14 sections, and 119 species [11]. However, there is still disagreement in the interspecies relationship of the genus *Camellia* [5].

The aforementioned classifications were based on morphological approach. Recent studies suggest that classifications purely based on the traditional morphological characteristics are insufficient [12], [13], [14]. Therefore, alternative taxonomic methods were developed for classification of *Camellia* [15], [16].

Contemporary advances in technology have resulted in new tools that allow classification based on alternative and innovative approaches. Lu et al. [12] used Fourier transform infrared spectroscopy (FTIR) on *Camellia* leaves to determine if they can be discriminated based on biochemical profiles. Chen et al. [3] and Yang et al. [17] used molecular approach based on genetic information for classification of *Camellia* species. Clearly, there is disagreement among researchers and no dominant method for this important classification problem has emerged. There are still many uncertainties about the relationships among species within sections and further taxonomic research on this section is necessary [13].

We acknowledge that although the flowers and the fruit are seasonal, the leaf lacks those limitations and their traits are more commonly used in plant taxonomic applications [18], [19], [20], [21]. Especially, Lin et al. [22] and Lu et al. [12] successfully revised three sections of genus *Camellia* based on leaf anatomic characters. Pi et al. [13] have used leaf morphology and anatomical characters for delimitation of species. They report that *“leaf features have been largely unexploited in taxonomic studies, resulting from a belief that they respond in a plastic manner to environmental forces.”*

Although leaf morphology has been the subject of some studies, lack of standard definitions of leaf characteristics has caused confusion in interpreting the value of the resulting classifications [13]. Taxonomical classification of *Camellia* based on a more comprehensive description of leaf morphology (also referred to as leaf architecture) is, therefore required. Leaf architecture refers to the placement and form of various elements constituting the outward expression of leaf structure, including leaf shape, leaf size, marginal configuration, gland position and venation pattern [23]. The leaf architecture has been the subject of several studies to resolve taxonomic and evolutionary relationships [24]. However, little research has been performed utilizing leaf architecture of genus *Camellia* species [25], [26], [27], [28].

The traditional analytical approaches employed by researchers to perform *Camellia* classification have included the principal component analysis, multivariate analysis, cluster analysis, and simulated annealing. Recently, some researchers have used supervised classification techniques in their studies. Supervised techniques are one of the most effective analysis tools in a variety of domains, such as information retrieval, remote sensing, and food bruise detection [29], [30], [31]. These tools apply available information about a category membership of samples to develop a model for classification of the genus. The classification model is developed using a training set with a priori defined categories and the performance is appraised using samples from a test set by comparing predicted categories with their true categories, as defined by experts [32], [33].

Artificial neural networks (ANN), as a pattern recognition tool, have been used for modeling complex systems [34], [35], [36], [37], [38]. Pandolfi et al. [15] discriminate and identify morphotypes of *Banksia integrifolia* by BP-ANN based on morphological and fractal parameters of leaves. Similarly, Pandolfi et al. [38] have used the BP-ANN approach to morphologically differentiate 17 Vietnamese tea plants. Support vector machine (SVM) is another supervised pattern recognition technology that has seen popularity of applications over the past several years [31], [39], [40], [41], [42]. This algorithm was developed in the machine learning community [43], [44] and is capable of learning in high-dimensional feature spaces [45].

Although pattern recognition tools have been applied in variety of fields, to the best of our knowledge this approach has not been used for classification of genus *Camellia* using leaf architecture data. We have used two different ANN architectures (LVQ-ANN and DAN2) and the support vector machine (SVM) to model *Camellia* classification. As stated earlier, there is still disagreement in the interspecies relationship of the genus *Camellia* [5], [12], [13], [14]. Researchers continue to use different taxonomical methods and analytical approaches to find more discriminating results. In this research, we combine the leaf architecture properties of genus *Camellia* with various pattern recognition tools, including a newly introduced method (DAN2), using a relatively large data set, to analyze the taxonomical classification of *Camellia* plants. The goal of the present work, therefore, is to classify *Camellia* species based on leaf architecture data. We present (*1*) results of using leaf morphological and venation characters of 93 species in five sections for *Camellia* classification, and (*2*) report the effectiveness of supervised pattern recognition techniques (LVQ-ANN, DAN2, and SVM) for such classifications and (*3*) compare their accuracy.

## Materials and Methods

### Materials

In this research we use comprehensive leaf morphology and leaf architecture for taxonomical classification of *Camellia*. Healthy leaf samples, consisting of 11 species from sect. *Furfuracea*, 16 species from sect. *Paracamellia*, 12 species from sect. *Tuberculata*, 34 species from sect. *Camellia*, and 20 species from sect. *Theopsis*, for a total of 93 plants, are examined in this study (following Chang [10] taxonomic treatment, Table S1). Leaf samples were taken from the third mature leaves that were fully exposed to sunlight and were horizontally arranged on the 2-year-old branches of the plants in the garden. At least three different individual plants per species are selected. Plant materials are all collected from the International *Camellia* Garden in Jinhua, Zhejiang Province (29°07′N, 119°35′E, altitude 40 m). Voucher specimens for all species are deposited in the Chemistry and Life Science College of Zhejiang Normal University (ZJNU) (see Appendix S1 for voucher details).

### Leaf veins specimen preparation

In order to use information from leaf vein patterns, we produced leaf veins specimens. The method used for making leaf veins specimen follows the process used by Zhang and Xia [46]. Leaves were placed in a glass tube, 10% sodium hydroxide (NaOH) was added in sufficient quantity to cover the material at 70–80°C for 3–4 hours. Since the leaf texture may differ among species, thicker leaves were treated for longer time periods. Leaves were taken out when the epidermis and mesophyll showed sufficient segregation. The leaves were then gently brushed to remove the epidermis and mesophyll with a paint brush. They were next rinsed in water and bleached by 10% hydrogen peroxide (H_2_O_2_) for approximately 60 minutes until the specimen was white. Bleached and cleared leaves were then washed in running water thoroughly. They were then fully stained by 0.5% methyl green for at least two hours. Subsequently, their pictures were taken with the Canon EOS 50D camera for further analysis.

### Leaf architecture data collection

(Table 1) presents list of the most commonly used leaf characteristics from literature [23], [26]. 31 characteristics of each leaf are collected and measured. All the test indexes were measured according to Hickey [23] specifications and guidelines. The leaf architecture data results are expressed as mean values. Below we describe the process in detail.

**Table 1:** Leaf architectural characters and related morphological characters [23], [26].

Characteristic	Encoding number					Figure 1
	0	1	2	3	4	
1. Whole lamina shape	Lanceolate	Ovate	Oblong	Longly oblong	Broadly oblong	See A
2. Base only	Auriculate	Rounded	Cuneate			See B
3. Apex	Long acuminate	Short acuminate	Obtusely acuminate	Acute		See C
4. Abaxial surface	Hairy	Glabrous				/
5. Adaxial surface	Hairy	Glabrous				/
6. Reticulate veins	Slight	Unconspicuous	Obvious	Conspicuous		See G
7. Secondary veins shape	Bend	Zigzag				See D
8. Secondary veins balance	Uniform	Uneven				See E
9. Areoles development	Incompletely	Imperfect				See K
10. Margin shape	Entire	Spinose	Cassidate	Setaceous	Spherulate	See H
11. Margin spacing	Regular	Irregular				See I
12. ASVPV on upper part^1^	Nearly right angle	Sharp angle				/
13. ASVPV on middle part	Nearly right angle	Sharp angle				/
14. ASVPV on lower part	Nearly right angle	Sharp angle				/
15. VADSV^2^	Nearly uniform	Upper secondary veins more obtuse than lower	Upper secondary veins more acute than lower			See F
16. Veinlets		Simple	1–2 times branched	More than 3 times branched		See J

1 ASVPV means angulation between secondary veins and primary veins;

2 VADSV means variations in angle of divergence of secondary veins.

### I. Leaf shape observation

**Figure 1:**
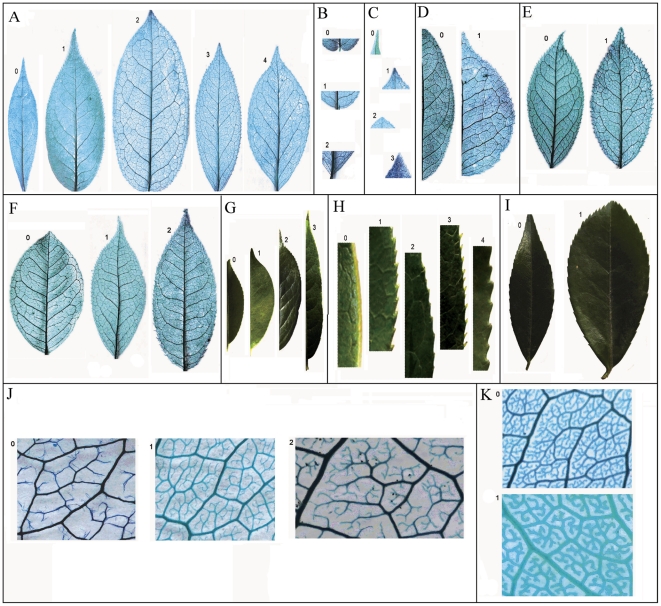
Leaf architectural characters and related morphological characters.

Following earlier research [23], [26], we selected 16 characteristics of leaf architecture and morphology that best describe leaf shapes for this research (Figure 1, Table 1). The same encoding values are used in all of the classification models.

### II. Leaf size measurements

The leaves of each species were scanned by CanoScan 4400FF Canon scanner (resolution of 4800*9600 dpi) using the WinFOLIA system (Regent Instruments Inc., Canada). For each sample, we measure leaf area, perimeter, vertical length, horizontal width, leaf aspect ratio (width/length), and leaf form factor (LEF). The formula used for estimating the LEF is:
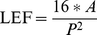
(1)Where *A* is the area and *P* is the perimeter. Other characteristics including number of secondary veins (pairs), petiole length, average value of entirely vein height (EVH), average value of leaf widest part height (LWPH), average ratio of EVH and leaf vertical length, average ratio of LWPH and leaf vertical length, serrulate length in upper part of leaf, serrulate length in middle part of leaf, and serrulate length in lower part of leaf, were measured with ImageJ Launcher (Broken Symmetry Software).

### Analysis methods

Traditional methods used for *Camellia* classification includes principal component analysis, multivariate analysis, cluster analysis, and simulated annealing. We examine the effectiveness of using various pattern recognition methods in this research. Specifically, we used a traditional artificial neural network (LVQ), a dynamic artificial neural network (DAN2), support vector machines (SVM), and cluster analysis for classification of the 93 samples. We used Chang (1998) classification data, presented in (Table S1), grouped into training and testing data sets, to measure and compare the accuracy of the three classification algorithms presented in this study. The training of these algorithms relies on (*a*) leaf characteristics data, and (*b*) class designation. The class designation used predefined Chang [10] classification.

#### LVQ-ANN classification model

**Figure 2:**
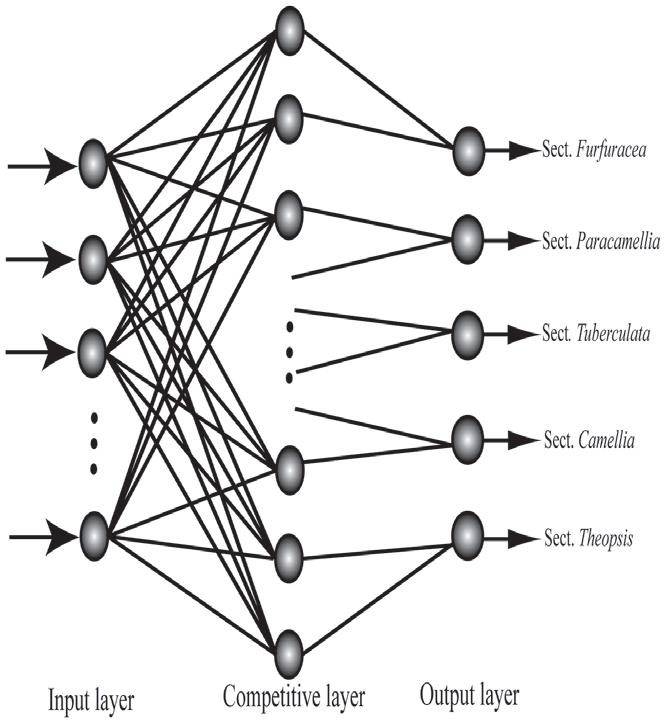
Schematic diagram of LVQ-ANN.

The first ANN model used is the Learning Vector Quantization (LVQ). LVQ is a special case of ANN that uses the “winner-take-all Hebbian learning strategy” [47], [48]. The network architecture consists of three layers: the input layer, the competitive layer (Kohonen layer) and the output layer. The input layer represents properties of species while the output layer represents the number of classes. In the competitive layer, each unit corresponds to a cluster, with the center designated as the “codebook” vector. An input vector closest to the codebook vector (using the Euclidean distance measure) belongs to the corresponding cluster. The optimal number of neurons in the competitive layer is determined experimentally. In this study, 93 samples belong to five different sections (categories) were selected: 48 samples were used to generate the classification model input for the training set and the remaining 45 samples were used in the testing stage (Table S1). Each vector of the input layer includes the 31 feature attributes of leaf architecture mentioned earlier. The number of nodes in the competitive layer varied from 20 to 30, and their impact was assessed on the respective classification capabilities. The output layer contained five neurons representing specific sections (taxon), including sect. *Furfuracea*, sect. *Paracamellia*, sect. *Tuberculata*, sect. *Camellia*, and sect. *Theopsis*. LVQ-ANN topology used in our study is shown in (Figure 2). By computing the Euclidean distance between a training vector and the weight of each node, the nearest node (‘winner node’) was generated. Winner nodes move towards the training vector when the winner nodes are in the same class, otherwise, they move away. The input vectors were then allocated to the category with the winning nodes. Training is complete when the mean square error (MSE) converges, or it is less than 0.1, or the number of training iterations reaches 1,000 epochs. We used two implementations of the LVQ algorithm: the LVQ1 and LVQ2. In LVQ1 a single best machine codebook vector is selected and moved closer or further for each data vector at each iteration, whereas in LVQ2 two sets of best machine codebook vectors are selected and only updated if one belongs to the desired class and one does not [49]. The LVQ-ANN modeling program was designed and programmed under MATLAB software (The Mathworks, Inc., Natick, MA, USA, version 7.9 R2009b).

#### DAN2 classification model

**Figure 3:**
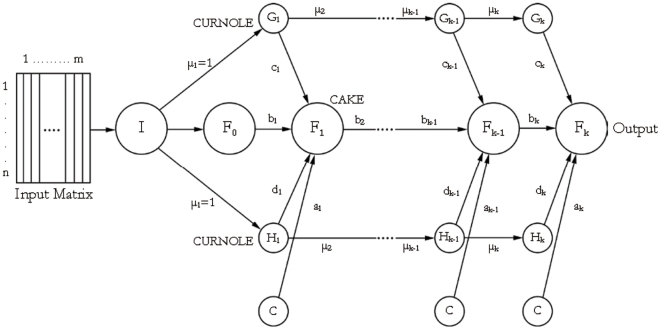
The DAN2 Network Architecture.

DAN2, (*A Dynamic Architecture for Artificial Neural Networks*), is a dynamic ANN model. It consists of input and output layers similar to LVQ and other ANNs. However, in DAN2 the number of hidden layers and hidden neurons are automatically and dynamically generated [50]. Two significant properties of DAN2 are: (*1*) its dynamic nature eliminates the need to experimentally define the number of hidden layers and hidden nodes, and (*2*) its architecture is fully scalable and can easily and effectively process any number of inputs. DAN2 is shown to be very effective in solving a variety of complex problems including classification problems [51], [52]. (Figure 3) presents the overall DAN2 architecture. As shown in (Figure 3), each hidden layer is composed of four nodes. The first node is the bias or constant (e.g. 1) input node, referred to as the C node. The second node is a function that encapsulates the “Current Accumulated Knowledge Element” (CAKE node) during the previous training step. The third and fourth nodes represent the current residual (remaining) nonlinear component of the process via a transfer function of a weighted and normalized sum of the input variables. Such nodes represent the “Current Residual Nonlinear Element” (CURNOLE nodes).

The scalability of DAN2 is a distinguishing strength of the approach from traditional artificial neural networks. In order to compare effectiveness of each technique, we use the exact same input vectors and the same training and testing data sets for the DAN2 model that were used in the LVQ models (Table S1) and report its results.

#### SVM classification model

SVM is based on statistical learning theory and structural risk minimization and was first proposed by Vapnik [44]. This approach generates hyperplanes to separate classes [53]. The boundaries of the hyperplane are represented by support vectors instead of a single boundary value. Support vectors run through the sample patterns which are the most difficult to classify and are closest to the actual class boundaries. Overfitting is prevented by specifying a maximum margin that separates the hyperplanes from the classes [54]. Samples violating this margin are penalized.

Once again, the exact same input vectors and training and test data sets that were used in LVQ were also used for the support vector machines models (Table S1). All C-SVM algorithms were implemented with LIBSVM (Version 3.0) under MATLAB software [55].

### Cluster analysis

Cluster analysis is the process of grouping data based on objects' attributes into similar and dissimilar groups. In this research, we use clustering analysis to classify 5 sections in genus *Camellia* based on the leaf architecture data (31 attributes) and to compare the results with Chang [10]. The clustering approach used is based on the Unweighted Pair-Group Method with Arithmetic Means (UPGMA). To address multidimensional scaling, the Gower General Similarity Coefficient is applied. The cluster analysis is conducted using MVSP software (Version 3.13n, Kovach Computing Services). The result of clustering analysis is presented in section 3.2.

## Results

### Leaf architecture data and related morphological data of samples

(Table S2) presents leaf architecture data for each *Camellia* species. The data shows that the leaf architecture for sect. *Furfuracea*, sect. *Paracamellia*, sect. *Tuberculata*, sect. *Camellia*, and sect. *Theopsis* are different. The most pronounced difference is in leaf vertical length (Table S2, column 20). Most vary from 5 to 10 cm; however, a few are closer to 15 cm (species in sect. *Furfuracea*), or less than 5 cm (sect. *Theopsis*). But for species in sect. *Paracamellia*, sect. *Tuberculata*, and sect. *Camellia*, the leaf vertical length values are diverse and vary widely. However, genus *Camellia* becomes a more natural group since it does have a series of common traits [6]. The common leaf architecture characteristics of the five sections are: leaf blade is symmetric, angulations between secondary veins and primary veins on upper part, on middle part, and on lower part is always at acute angle (Table S2, column 11–13), veinlets are 1–2 times branched (Table S2, column 16), and areoles development is incomplete (Table S2, column 17). As shown in (Table S2, columns 6–9, 14–15), there are differences in leaf venation characteristics such as the reticulate veins (column 6), margin shape (column 7), margin spacing (column 8), secondary veins shape (column 9), the number of secondary veins variations in angle of divergence between primary and secondary veins (columns 14), number of secondary veins (column 15).

### Cluster analysis based on leaf architecture data

**Figure 4:**
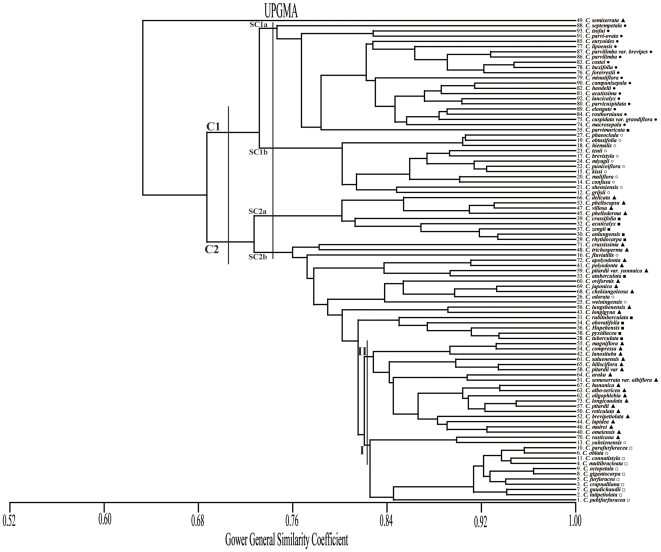
UPGMA dendrogram of genus *Camellia* based on leaf architectural characteristics. sect. *Camellia* (▴), sect. *Theopsis* (•), sect. *Tuberculata* (▪), sect. *Paracamellia* (○), sect. *Furfuracea* (□).

The dendrogram resulting from hierarchical cluster analysis grouped the 93 species into two main clusters (Figure 4). Cluster 1 (C1), included all species of sect. *Theopsis*, and most species of sect. *Paracamellia*, Cluster 2 (C2) had all species of sect. *Furfuracea*, sect. *Tuberculata*, and most species of sect. *Camellia*. On closer inspection, C1 contained two subclusters: subcluster 1a (SC1a) comprised of all special species of sect. *Theopsis*, *C. semiserrata* which belongs to sect. *Camellia*, and *C. parvimurivata* which belongs to sect. *Tuberculata*. Subcluster 1b (SC1b) comprised of all species of *Paracamellia*. C2 contained the remaining three sections and differed from previous classification [10]. For instance, subcluster 2a (SC2a) and subcluster 2b (SC2b) are mainly comprised of sect. *Camellia* and sect. *Tuberculata*, indicating significant closeness with species affinities. Therefore, we suggest that sect. *Camellia* and sect. *Tuberculata* may be merged into one section. However, branch I (I) and branch II (II) were clearly divided by species of sect. *Furfuracea* and some species of sect. *Camellia*. These results provide mostly the same categorization of genus *Camellia* as specified by Chang [10].

### LVQ-ANN, DAN2, and SVMs classification based on leaf architecture data

(Table 2) shows the classification results for the Learning Vector Quantization neural network (LVQ-ANN) with different number of competitive layer neurons. Following suggestion of other researchers [32], and in order to achieve the best performance for the LVQ-ANN algorithms, we experimented by varying the number of neurons in the competitive layer. Both the LVQ1 and LVQ2 learning algorithms reached their highest classification accuracy with the competitive layer neuron number set to 24 and 25, respectively. Comparing the two LVQ-ANN methods, revealed that LVQ1 learning algorithm produces a more accurate results, for both the training and testing data sets (75% and 60%), than LVQ2 (54.17% and 55.56%) (Table 3). Although, the classification of sect. *Theopsis* by LVQ2-ANN reached 100.00% accuracy, when the number of competitive layer neuron was 20, 24, or 27 (Table 2); overall, the classification results produced with LVQ1 learning algorithm were more stable, especially in the sect. *Theopsis* classification (accuracy of 90.00%), and the sect. *Paracamellia* (accuracy of 37.50%) (Table 3). Although LVQ-ANN does not provide acceptably accurate results for this data set, the advantage of this model is in its simplicity and the fact that the input data does not need to be normalized or orthogonalized. Thus, LVQ-ANN may be used as a simple control method for classification.

**Table 2:** The classification results of LVQ-ANN with different number of competitive layer neuron based on LVQ1 and LVQ2 learning algorithm.

Samples	Sample numbers	Learning algorithm	Identification rate of different numbers of competitive layer neuron numbers										
			20	21	22	23	24	25	26	27	28	29	30
**Sect. ** ***Furfuracea***	5	LVQ1	80.00%	20.00%	80.00	20.00%	60.00%	20.00%	40.00%	60.00%	20.00%	40.00%	20.00%
		LVQ2	0.00%	0.00%	0.00%	80.00	0.00%	80.00%	80.00%	0.00%	0.00%	20.00%	20.00%
**Sect. ** ***Paracamellia***	8	LVQ1	37.50%	37.50%	37.50%	37.50%	37.50%	37.50%	37.50%	37.50%	37.50%	37.50%	37.50%
		LVQ2	0.00%	75.00%	75.00%	75.00%	0.00%	75.00%	75.00%	0.00%	75.00%	75.00%	75.00%
**Sect. ** ***Tuberculata***	6	LVQ1	0.00%	16.67%	16.67%	16.67%	16.67%	16.67%	16.67%	0.00%	16.67%	0.00%	16.67%
		LVQ2	0.00%	0.00%	0.00%	0.00%	0.00%	0.00%	0.00%	0.00%	0.00%	0.00%	0.00%
**Sect. ** ***Camellia***	16	LVQ1	62.50%	68.75%	62.50%	68.75%	68.75%	68.75%	68.75%	68.75%	62.50%	62.50%	62.50%
		LVQ2	75.00%	75.00%	87.50%	68.75%	81.25%	93.75%	81.25%	81.25%	81.25%	75.00%	75.00%
**Sect. ** ***Theopsis***	10	LVQ1	90.00%	90.00%	90.00%	90.00%	90.00%	90.00%	90.00%	90.00%	90.00%	90.00%	90.00%
		LVQ2	100%	0.00%	0.00%	0.00%	100%	0.00%	0.00%	100%	0.00%	0.00%	0.00%
Total accuracy (%)		LVQ1	57.78%	55.56%	60.00%	55.56%	60.00%	55.56%	57.78%	57.78%	53.33%	53.33%	53.33%
		LVQ2	48.89%	40.00%	44.44%	46.67%	51.11%	55.56%	51.11%	51.11%	42.22%	48.89%	42.22%

**Table 3:** Comparing best classification accuracy of LVQ1, LVQ2, DAN2 and RBF-SVM.

Class	LVQ1 Training	LVQ1 Testing	LVQ2 Training	LVQ2 Testing	DAN2 Training	DAN2 Testing	SVM Training	SVM Testing
**Sect. ** ***Furfuracea***	83.33% (5/6)	80% (4/5)	33.33% (2/6)	80% (4/5)	100% (6/6)	100% (5/5)	100% (6/6)	100% (5/5)
**Sect. ** ***Paracamellia***	62.5% (5/8)	37.50% (3/8)	75% (6/8)	75.00% (6/8)	100% (8/8)	87.50% (7/8)	100% (8/8)	100% (8/8)
**Sect. ** ***Tuberculata***	16.67% (1/6)	16.67% (1/6)	0% (0/6)	0% (0/6)	83.33% (5/6)	66.70% (4/6)	83.33% (5/6)	83.33% (5/6)
**Sect. ** ***Camellia***	94.44% (17/18)	62.50% (10/16)	100% (18/18)	93.75% (15/16)	100% (18/18)	93.75% (15/16)	100% (18/18)	100% (16/16)
**Sect. ** ***Theopsis***	80.00% (8/10)	90.00% (9/10)	0% (0/10)	0% (0/10)	100% (10/10)	100% (10/10)	100% (10/10)	100% (10/10)
Total Accuracy (%)	75.00% (36/48)	60.00% (27/45)	54.17% (26/48)	55.56% (25/45)	97.92% (47/48)	91.11% (41/45)	97.92% (47/48)	97.78% (44/45)

DAN2 is a dynamic neural network model that does not require model configuration or parameter optimization. DAN2's algorithm, at every iteration, solves a nonlinear minimization problem. Specifically, the nonlinear optimization strategy used in DAN2 estimates a nonlinear parameter. Like all nonlinear optimization methods for non-convex/non-concave functions, obtaining global optimization is never guaranteed. Similar to other optimization applications, choice of a good starting point can improve convergence to local optimum and beginning the search at various starting points can facilitate reaching multiple local optima. Ghiassi and Saidane [50] identify the starting point as F_0_(X) and use the training data and the standard linear regression to obtain its value. In classification problems, F(X) only takes binary (or integer) values, so in addition to the standard MLR; we have experimented with using a rudimentary kNN solution to obtain a good starting point. The kNN approach used is a simplified method that only considers one or two values for k (k = 1 or k = 3) to quickly obtain a starting point value. In this study, we use the exact same data sets used in the LVQ model to train DAN2 models. During DAN2 training, we iteratively reduce training error tolerance by specifying a SSE/MSE value. The model training stops either when it reaches this value or a predefined number of iterations. The value of this error level can be iteratively reduced to a desired level. The model uses internally defined metrics to avoid overfitting [51]. DAN2 model uses the “one-vs.-all” classification approach detailed in [53]. Results from this model are presented in (Table 3). The overall training and testing accuracy for this model is 97.92% and 91.11% respectively (sect. *Furfuracea*-100%, sect. *Paracamellia*-87.5%, sect. *Tuberculata*-66.67%, sect. *Camellia*-93.75%, and sect. *Theopsis*-100%, respectively, for the test data set). DAN2 model presents better results than LVQ models and does not require model configurations.

**Figure 5:**
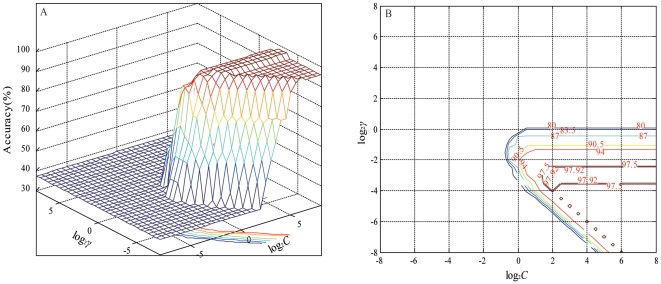
Classification accuracy in different kernel parameter (*C*) and regularization parameter (*γ*) by cross-validation. Three dimension diagram (A) and contour map (B).

**Figure 6:**
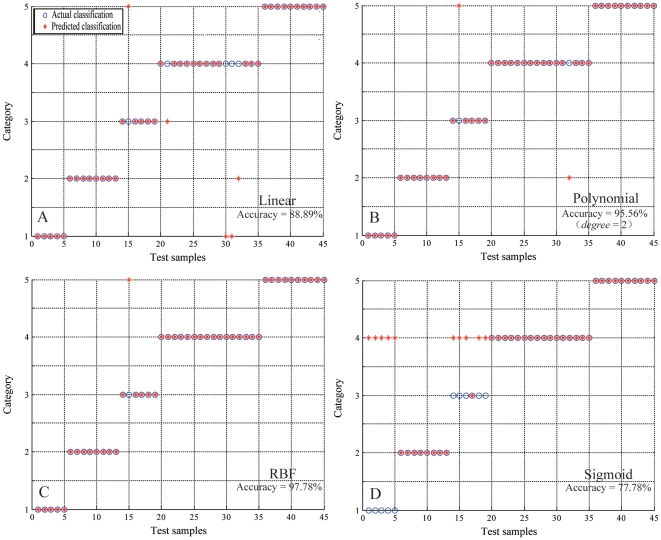
The classification results of linear, polynomial, RBF and sigmoid SVMs with the optimal parameters.

We next present results of using SVM for this analysis. To use the support vector machines (SVMs) model, and in order to obtain the best performance, the two SVM parameters of regularization (*C*) and kernel parameter (*γ*) are optimized using cross validation. Linear, polynomial, sigmoid, and radial basis function (RBF) kernel classifiers were tested in this study. We used the same input vectors, training and testing data sets for the LVQ models and the SVM models. As seen in (Figure 5), the highest accuracy of 97.92% was achieved when *C* = 2.828 and *γ* = 0.088 for the training data set. All SVM models are optimized by manipulating *C* and *γ* parameters to obtain the best training accuracy. (Figure 6) presents the classification results of all the SVM models, with optimal parameters for the test data sets. The linear kernel overall accuracy for the test data set is 88.89%. For the polynomial model, the degree parameter (*d*) ranges from three to ten. (Table 4) shows the classification results of polynomial SVM models with different degrees. The best results, 95.56% accuracy for the test data set, was obtained for *d* = 3. The polynomial SVM classifier with polynomial degree *d* = 7 had a classification rate of 88.89% for the test data set, which was similar to the linear model. (Table 4) also shows that classification accuracies do not improve for the polynomial degree larger than three. The sigmoid model performed less accurately than other SVM models. The overall accuracy for the test data for this model was only 77.78%. (Figure 6) shows that the RBF SVM classifier offers the best results with overall accuracy of 97.78% for the test data set (sect. *Furfuracea*-100%, sect. *Paracamellia*-100%, sect. *Tuberculata*-83.33%, sect. *Camellia*-100%, and sect. *Theopsis*-100%, respectively, for the test data sets).

**Table 4:** The classification results of the polynomial SVM with different degrees in the optimal parameters (C = 2.828, γ = 0.088).

Samples	Sample Numbers	Identification rate of polynomial classifiers in different degree							
		3	4	5	6	7	8	9	10
**Sect. ** ***Furfuracea***	5	100%	100%	100%	100%	100%	100%	100%	100%
**Sect. ** ***Paracamellia***	8	100%	87.50%	87.50%	87.50%	75.00%	75.00%	87.50%	87.50%
**Sect. ** ***Tuberculata***	6	83.33%	83.33%	83.33%	83.33%	83.33%	83.33%	66.67%	66.67%
**Sect. ** ***Camellia***	16	93.75%	93.75%	93.75%	93.75%	87.50%	81.25%	75.00%	68.75%
**Sect. ** ***Theopsis***	10	100%	100%	100%	100%	100%	100%	100%	100%
Total accuracy (%)		95.56%	93.33%	93.33%	93.33%	88.89%	86.67%	84.44%	82.22%

## Discussion

**Figure 7:**
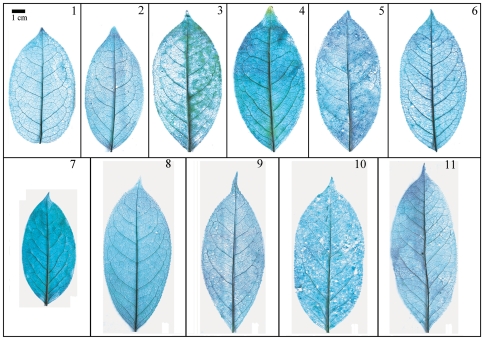
Leaf specimen of sect. *Furfuracea*. Numbers in figure corresponding species numbers in Table S1. Scale bar = 1 cm.

Taxonomical classification based on description of leaf morphology is an effective approach [13]. Leaf architecture has been the subject of several studies in taxonomy and evolutionary relationships of taxa with controversial genera [24]. The architectural properties of leaf venation patterns for systematic classification have also been studied [56], [57], [58]. Macrofossils studies have shown that the leaf venation patterns can be extensively utilized in identifying fossil taxa in palaeobotany [59]. The lamina morphological and venation character details of *Camellia* are also shown in the Figure 7 through [Fig pone-0029704-g008] [Fig pone-0029704-g009] [Fig pone-0029704-g010] [Fig pone-0029704-g011] [Fig pone-0029704-g012] [Fig pone-0029704-g013] [Fig pone-0029704-g014] [Fig pone-0029704-g015] [Fig pone-0029704-g016] 17. We found significant results using the leaf venation pattern for identifying various *Camellia* species indicating the importance of this tool for classification.

**Figure 8 pone-0029704-g008:**
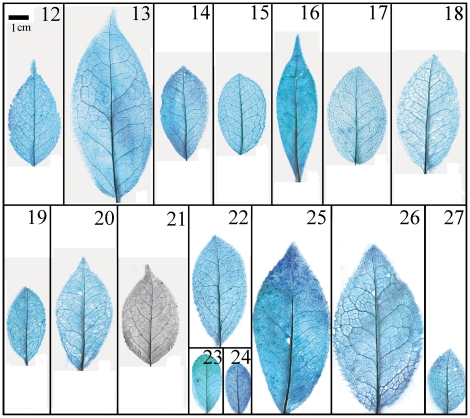
Leaf specimen of sect. *Paracamellia*. Numbers in figure corresponding species numbers in Table S1. Scale bar = 1 cm.

**Figure 9 pone-0029704-g009:**
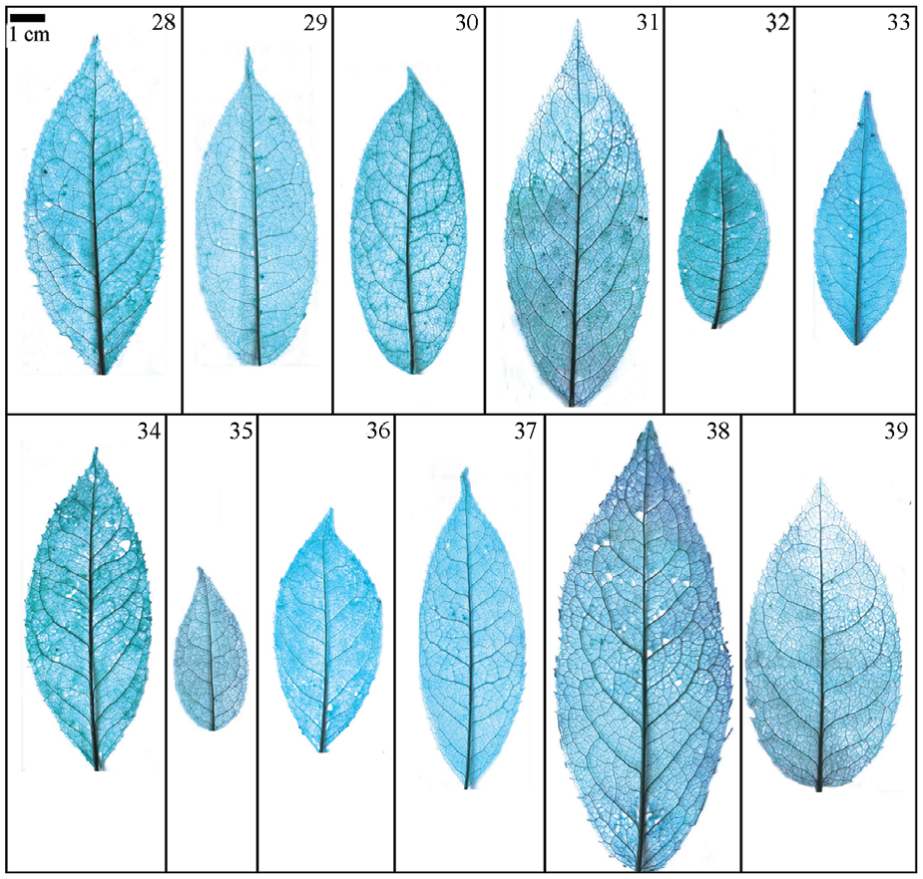
Leaf specimen of sect. *Tuberculata*. Numbers in figure corresponding species numbers in Table S1. Scale bar = 1 cm.

**Figure 10 pone-0029704-g010:**
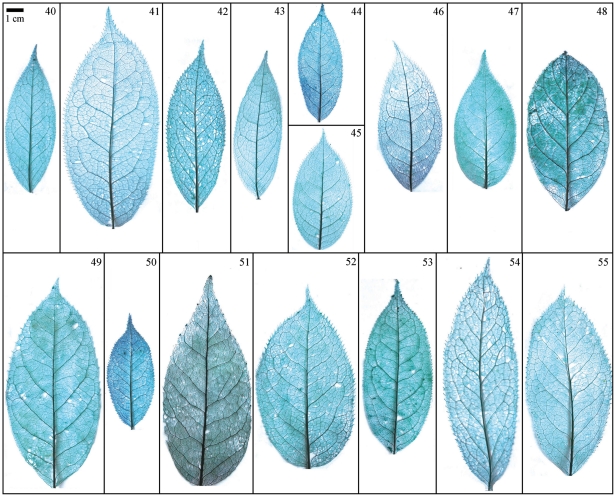
Leaf specimen of sect. *Camellia* (No. 40–55). Numbers in figure corresponding species numbers in Table S1. Scale bar = 1 cm.

**Figure 11 pone-0029704-g011:**
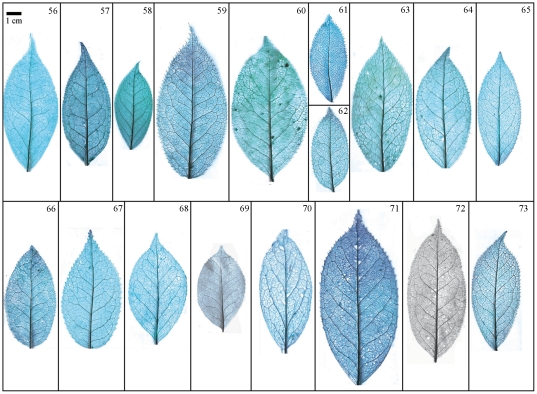
Leaf specimen of sect. *Camellia* (No. 56–73). Numbers in figure corresponding species numbers in Table S1. Scale bar = 1 cm.

**Figure 12 pone-0029704-g012:**
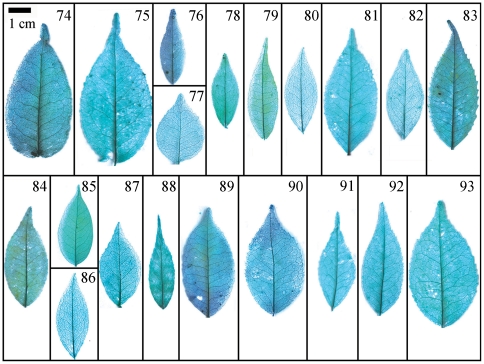
Leaf specimen of sect.*Theopsis*. Numbers in figure corresponding species numbers in Table S1. Scale bar = 1 cm.

**Figure 13 pone-0029704-g013:**
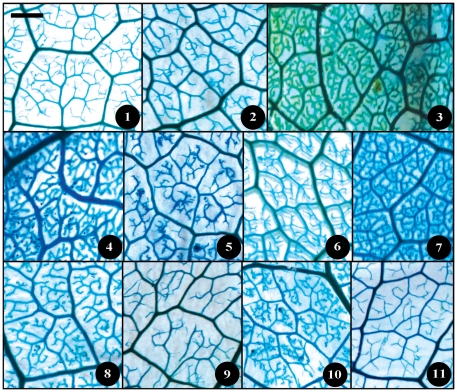
Detail venation characters of sect. *Furfuracea*. Numbers in figure corresponding species numbers in Table S1. Scale bar = 1 mm.

**Figure 14 pone-0029704-g014:**
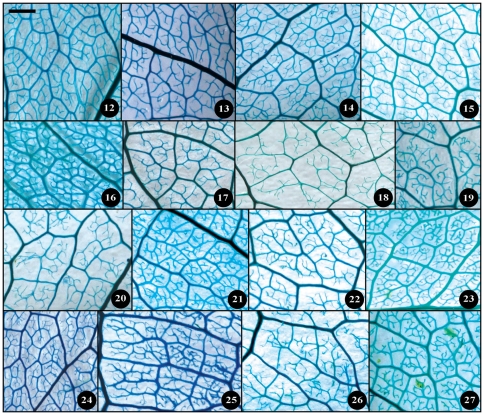
Detail venation characters of sect. *Paracamellia*. Numbers in figure corresponding species numbers in Table S1. Scale bar = 1 mm.

**Figure 15 pone-0029704-g015:**
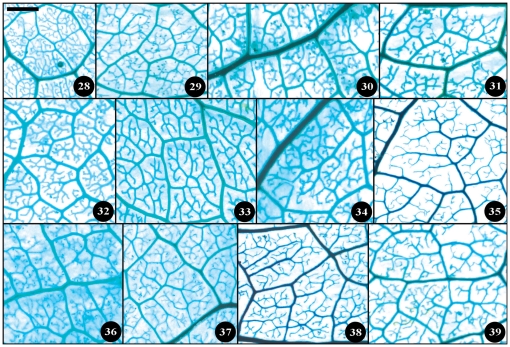
Detail venation characters of sect. *Tuberculata*. Numbers in figure corresponding species numbers in Table S1. Scale bar = 1 mm.

**Figure 16 pone-0029704-g016:**
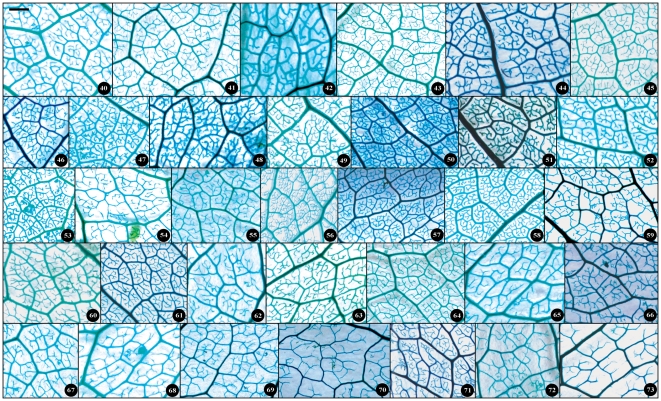
Detail venation characters of sect. *Camellia*. Numbers in figure corresponding species numbers in Table S1. Scale bar = 1 mm.

**Figure 17:**
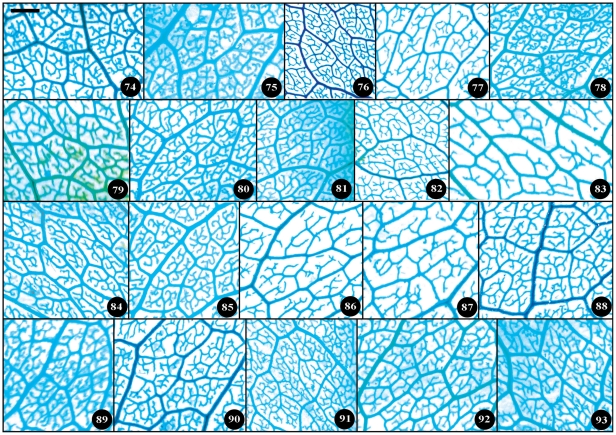
Detail venation characters of sect. *Theopsis*. Numbers in figure corresponding species numbers in Table S1. Scale bar = 1 mm.

To identify and distinguish *Camellia* plants, the floras edited by botanists such as Chang [10] and Ming [6] are commonly used as a comprehensive resource [60]. Indented dichotomous keys in the literature are commonly used as the identification keys. When a new unknown species needs to be classified, we always turn to these floras, and the identification process often follows a predefined path with the observed characteristics. However, since the traditional information retrieval processes are tedious, the final classification is often subjective. Clustering and pattern recognition techniques, especially DAN2 and SVM, used in this research, are shown to be an effective and objective classification tools that can be used to classify new species. We present results from using these tools along with the leaf architecture data for classifying 93 *Camellia* species (Table S2).

The classification of species from our dendrogram is mostly in agreement with previous research, indicating that the discrimination of these species by leaf architecture data reflects their phylogenetic relations. In this discussion, we compare and contrast our results from applying cluster analysis and pattern recognition methods using leaf architecture-based data, with existing classifications. Specifically, we compare results from the cluster analysis, and the two pattern recognition methods with the best results (DAN2, and the RBF-SVM) with those of Chang [10] and Ming [6].

Analysis of leaf characters data has been successfully employed to investigate plant taxonomy. Our study suggests that leaf architecture-based *Camellia* classification using pattern recognition techniques can be used to discriminate plants at the genus level. In this study, the results of cluster analysis using leaf architecture data mainly support Chang's [10] classification of *Camellia*. However, our results continue to strengthen the controversy about a number of species.

The separation of 93 species in the dendrogram obtained in this study using clustering analysis (Figure 4) was mostly in agreement with the taxonomy of Chang [10]. However, as illustrated in (Figure 4), *C. weiningensis* (No. 25) has similar attributes with species belonging to sect. *Camellia*. This finding makes it reasonable to merge *C. weiningensis* into sect. *Camellia*; thus, validating Ming's [6] classification of the *C. weiningensis*. Similarly, Chang [10] classifies *C. semiserrata* (No. 49) to belong to sect. *Camellia*, *and C. parvimuricata* (No. 35) to belong *to* sect. *Tuberculata*. We find these two species (Nos. 35 & 49) to have closer relationship with sect. *Theopsis*. Therefore, we find it more reasonable to merge them into sect. *Theopsis*. In addition, Chang's taxonomic treatment advocates sect. *Tuberculata* and sect. *Camellia* as two independent sets. However, as depicted in (Figure 4), species of sect. *Tuberculata* are closer to sect. *Camellia*. We recommend that they should be merged into one section. For sect. *Furfuracea*, all species are grouped together, validating Chang's taxonomic treatment. We disagree with Ming's [6] suggestions that sect. *Furfuracea* should be canceled and that its species arrangements should be adjusted. Studies of Ming [6] suggest that the *C. hiemalis* (No. 18) species should be classified as a variant of *C. sasanqua* (belonging to sect. *Oleifera*), whereas our hierarchical dendrogram based on leaf architecture data shows *C. hiemalis* to be similar to the species of sect. *Paracamellia* and does not support merging of *C. hiemalis* into *C. sasanqua*. Our findings support Chang's [10] treatments of these two species. Moreover, *C. oblate* (No. 6) and *C. parafurfuracea* (No. 10) are classified as one species class by Ming [6]. Our study shows that the bases of *C. oblate* and *C. parafurfuracea* are round and both species have similar leaf architecture characteristics. Our cluster analysis reaffirms Ming's treatment of these two species so it is reasonable to consider *C. oblate* and *C. parafurfuracea* as one species. The two species *C. parvilimba var. brevipes* (No. 87) *and C. parvilimba* (No. 86) are very similar, indicating a high degree of homogeneity. For these species, we agree with Chang in considering *C. parvilimba var. brevipes* as a variety of *C. parvilimba*. These results augment the usefulness of leaf architecture data for plant taxonomic treatments. We also note that deviation from the classification needs to be further investigated to see if a misclassification is due to the underlying algorithm's fitting of the data or Chang's [10] designation of the species.

In analyzing results from the pattern recognition techniques, we note that although LVQ-ANN did not produce very accurate results, when comparing this approach with other ANNs, LVQ has the advantage that it can classify any set of input vectors, has a fast learning algorithm [61] and is used extensively in the literatures [62], [63], [64], [65], [66], [67], [68].

In analyzing DAN2 results (Table 3), we note that all species of sect. *Furfuracea* and *Sect. Theopsis* conform to Chang's classifications. In sect. *Paracamellia*, DAN2 suggests the *C.winingensis* (No. 25) species to belong to sect. *Camellia*. This result is similar to the clustering algorithm's results and we disagree with Chang's classification. We suggest *C.winingensis* to belong to sect. *Camellia*, and agree with Ming's taxonomic results. Additionally, our cluster analysis show that sect. *Camellia* and sect. *Tuberculata* have significant closeness with species affinities. DAN2 results classify *C. hupehensis* (No. 36), *C. zengii* (No. 37) and *C. crassifolia* (No. 39) species to belong to sect. *Camellia*. This conclusion validates Chang's view about the close evolutionary relationship between sect. *Camellia* and sect. *Tuberculata*. Furthermore, this shows that *C. hupehensis*, *C. zengii* and *C. crassifolia* may indeed have underlying links in biological evolutionary principles with species of sect. *Camellia*. This finding emphasizes the need for further research in this branch.

In analyzing the classification results from the SVM approach (Figure 6), we note that the *C. fluviatilis* (No. 16) in sect. *Paracamellia* was incorrectly identified by all SVM classifiers. This specie was incorrectly identified as sect. *Theopsis* by linear, polynomial (*d* = 2), and RBF classifiers and as sect. *Camellia* by sigmoid classifier. The results suggest *C. fluviatilis* to be similar to the species of sect. *Theopsis* or sect. *Camellia*. We also note that in the clustering analysis, (Figure 4) shows *C. fluviatilis* to have closer relation with sect. *Camellia*. Therefore, it may be more reasonable to merge it into sect. *Camellia* rather than merging it into sect. *Paracamellia* as suggested by Chang [10]. Finally, the RBF-SVM classifier offers the best conformance to Chang's classification, validating its effectiveness as a classification tool for plants.

In general, for this data set, the SVM approach shows better generalization than LVQ-ANN and DAN2. As pointed out by Pandolfi et al. [38], success of ANN methods usually depends on the quantity, validity, and accuracy of training data. However, other researchers have shown SVM to perform well for ill-posed problems with few training records [45], [69], [70], [71]. Our results confirm this property of SVM. The RBF-SVM kernel used in this study offers the best results by conforming to Chang [10] classification. However, it should be noted that using Chang's classification as a reference is controversial and literature suggests variation from this classification. Although DAN2 displayed lower classification accuracy in conformance to Chang's, we cannot dismiss the correctness of its results. Taxonomy is a dynamic field and existing theory does not support 100% accuracy of any classification due in part to the fact that taxonomic treatments based on different features may generate different results. Therefore, it should not be surprising to see some divergences among different tools such as those observed in using DAN2 and RBF-SVM models in comparison with *Camellia* taxonomic systems of Chang [10] and Ming [6]. Overall results from using the leaf architecture data combined with pattern recognition and discrimination methods (LVQ-ANN, DAN2, and SVM), is shown to be an effective tool for identification of genus *Camellia*.

### Conclusion

In conclusion, lamina morphological and venation characters of 93 species in five sections (sect. *Furfuracea*, sect. *Paracamellia*, sect. *Tuberculata*, sect. *Camellia*, and sect. *Theopsis*) are reported. The hierarchical dendrogram based on leaf architecture data confirms the morphological classification of the five sections proposed by Chang's taxonomic treatment. LVQ-ANN, DAN2, and SVMs models based on the 31 leaf architecture attributes were constructed. In LVQ-ANN models, the best classification accuracy is 60.00% for the test data set when number of competitive layer neuron is 22 or 24 using the LVQ1 learning algorithm. The best DAN2 model offers a classification accuracy of 91.11% for the test data. In SVM models, the best classification accuracy is 97.78% using the RBF SVM classifier with *C* = 2.828 and *γ* = 0.088. The overall results indicate that leaf architecture analysis using pattern recognition tools, especially DAN2 and SVM algorithms, can be effectively used to distinguish the *Camellia* genus and other plant taxa.

## Supporting Information

Appendix S1 Collection localities and vouchers of studied specimens.
(DOC)

Table S1 Species studied, as classified by Chang (1998).
(DOC)

Table S2 Data matrix of characteristics on leaf architecture of genus *Camellia*.
(DOC)
