# Supplementary material for: Classification of Camellia (Theaceae) Species Using Leaf Architecture Variations and Pattern Recognition Techniques
Source: PLoS One. 2012 Jan 3;7(1):e29704. doi: 10.1371/journal.pone.0029704 (PMC3250490; doi:10.1371/journal.pone.0029704)
Supplement: Appendix S1 — Collection localities and vouchers of studied specimens. (DOC) [file pone.0029704.s001.doc]

**Appendix S1.** Collection localities and vouchers of studied specimens.

| Taxon,; vouchers; accession number (all specimens from Jinhua International *Camellia* Species Garden, Zhejiang, China) |
| --- |
| *C. pubifurfuracea*; *H.E. Tian*; 200610170901 (ZJNU). *C. latipetiolata*; *H.E. Tian*; 200610171001 (ZJNU). *C. crapnalliana*; *H.E. Tian*; 200610171101 (ZJNU). *C. multibracteata*; *H.E. Tian*; 200610171201 (ZJNU). *C. furfuracea*; *H.E. Tian*; 200610171301 (ZJNU). *C. oblate*; *Q.F. Peng*; 200610221401 (ZJNU). *C. gaudichaudii*; *H.E. Tian*; 200610171501 (ZJNU). *C. gigantocarpa*; *Q.F. Peng*; 200610221601 (ZJNU). *C. octopetala*; *Q.F. Peng*; 200610221701 (ZJNU). *C. parafurfuracea*; *H.E. Tian*; 200610171801 (ZJNU). *C. connatistyla*; *H.E. Tian*; 200610171901 (ZJNU). *C. grijsii*; *J.B. Shen*; 200701122001 (ZJNU). *C. yuhsienensis*; *J.B. Shen*; 200701122102 (ZJNU). *C. confusa*; *J.B. Shen*; 200612232201 (ZJNU). *C. kissi*, *J.B. Shen*; 200701122302 (ZJNU). *C. fluviatilis*; *X.Y. Lin*; 200701282401 (ZJNU). *C. brevistyla*; *J.B. Shen*; 200612232501 (ZJNU). *C. hiemalis*; *J.B. Shen*; 200612232602 (ZJNU). *C. obtusifolia*; *J.B. Shen*; 200701122701 (ZJNU). *C. maliflora*; *J.B. Shen*; 200701122802 (ZJNU). *C. shensiensis*; *J.B. Shen*; 200701122901 (ZJNU). *C. puniceiflora*; *J.B. Shen*; 200701123001 (ZJNU). *C. tenii*; *X.Y. Lin*; 200701283102 (ZJNU). *C. miyagii*; *J.B. Shen*; 200701123001 (ZJNU). *C. weiningensis*; *S.S. Hong*; 2011040139 (ZJNU). *C. odorata*; *X.Y. Lin*; 20070128350 (ZJNU). *C. phaeoclada*; *J.B. Shen*; 200701123602 (ZJNU). *C. tuberculata*; *Q.F. Peng*; 05112301 (ZJNU). *C. rhytidocarpa*; *B. Jiang*; 06111201 (ZJNU). *C. anlungensis*; *Q.F. Peng*; 05112302 (ZJNU). *C. rubituberculata*; *B. Jiang & Q.F. Peng*; 06062302 (ZJNU). *C. acuticalyx*; *B. Jiang & Q.F. Peng*; 06062304 (ZJNU). *C. atuberculata*; *B. Jiang & Q.F. Peng*; 06090401 (ZJNU). *C. obovatifolia*; *B. Jiang & Q.F. Peng*; 06062305 (ZJNU). *C. parvimuricata*; *B. Jiang & Q.F. Peng*; 06090404 (ZJNU). *C. Hupehensis*; *Q.F. Peng*; 05112303 (ZJNU). *C. zengii*; *B. Jiang & Q.F. Peng*; 06061201 (ZJNU). *C. pyxidiacea*; *B. Jiang & Q.F. Peng*; 05112304 (ZJNU). *C. crassifolia*; *S.S. Hong*; 2011040104 (ZJNU). *C. omeiensis*; *Q.F. Peng*; 200603611 (ZJNU). *C. polyodonta*; *Q.F. Peng*; 200603621 (ZJNU). *C. lanosituba*; *Q.F. Peng*; 20060363 (ZJNU). *C. longigyna*; *Q.F. Peng*; 200603641 (ZJNU). *C. lapidea*; Q. F. Peng 200603651 (ZJNU). *C. phelloderma*; *Q.F. Peng*; 200603661 (ZJNU). *C. mairei*; *Q.F. Peng*; 200603671 (ZJNU). *C. villosa*; *Q.F. Peng*; 200603681 (ZJNU). *C. trichosperma*; *Q.F. Peng & X.Y. Lin*; 200604691 (ZJNU). *C. semiserrata*; *Q.F. Peng & X.Y. Lin*; 200604701 (ZJNU). *C. reticulata*; *Q.F. Peng & X.Y. Lin*; 200604721 (ZJNU). *C. semiserrata var. albiflora*; *Q.F. Peng & X.Y. Lin*; 200604721 (ZJNU). *C. brevipetiolata*; *Q.F. Peng*; 200603731 (ZJNU). *C. phellocapsa*; *Q.F. Peng*; 200603742 (ZJNU). *C. compressa*; *Q.F. Peng*; 200603751 (ZJNU). *C. magniflora*; *Q.F. Peng*; 200603761 (ZJNU). *C. lungshenensis*; *Q.F. Peng & B. Jiang*; 200610771 (ZJNU). *C. pitardii*; *Q.F. Peng & B. Jiang*; 200610801 (ZJNU). *C. pitardii var. alba*; *Q.F. Peng*; 200603811 (ZJNU). *C. pitardii var. yunnaica*; *Q.F. Peng*; 200603821 (ZJNU). *C. oviformis*; *Q.F. Peng*; 200603861 (ZJNU). *C. saluenensis*; *Q.F. Peng & B. Jiang*; 200610901 (ZJNU). *C. oligophlebia*; *Q.F. Peng*; 200603931 (ZJNU). *C. albo-sericea*; *Q.F. Peng*; 200603911 (ZJNU). *C. uraku*; *Q.F. Peng*; 200603941 (ZJNU). *C. hilisciflora*; *Q.F. Peng*; 2006031001 (ZJNU). *C. delicata*; *Q.F. Peng*; 2006031041 |

**Appendix S1.** Continued

| Taxon,; vouchers; accession number (all specimens from Jinhua International *Camellia* Species Garden, Zhejiang, China) |
| --- |
| (ZJNU). *C. hunanica*; *Q.F. Peng & B. Jiang*; 2006101051 (ZJNU). *C. chekiangoleosa*; *Q.F. Peng & B. Jiang*; 2006101111 (ZJNU). *C. japonica*; *Q.F. Peng & B. Jiang*; 2006101131 (ZJNU). *C. rusticana*; *Q.F. Peng & B. Jiang*; 2006101141 (ZJNU). *C. crassissima*; *Q.F. Peng & X.Y. Lin*; 2006041181 (ZJNU).*C. apolyodonta*; *Q.F. Peng & X.Y. Lin*; 2006041191 (ZJNU). *C. longicaudata*; *S.S. Hong*; 2011040138 (ZJNU). *C. macrosepala*; *S.S. Hong*; 2011040107 (ZJNU). *C. cuspidata var. grandiflora*; *S.S. Hong*; 2011040108 (ZJNU). *C. forerrestii*; *S.S. Hong*; 2011040109 (ZJNU). *C. lipoensis*; *S.S. Hong*; 2011040110 (ZJNU). *C. buxifolia*; *S.S. Hong*; 2011040111 (ZJNU). *C. minutiflora*; *S.S. Hong*; 2011040112 (ZJNU). *C. parvicuspidata*; *S.S. Hong*; 2011040113 (ZJNU). *C. acutissima*; *S.S. Hong*; 2011040114 (ZJNU). *C. handelii*; *S.S. Hong*; 2011040115 (ZJNU). *C. costei*; *S.S. Hong*; 2011040116 (ZJNU). *C. rosthorniana*; *S.S. Hong*; 2011040117 (ZJNU). *C. euryoides*; *S.S. Hong*; 2011040118 (ZJNU). *C. parvilimba*; *S.S. Hong*; 2011040119 (ZJNU). *C. parvilimba var. brevipes*; *S.S. Hong*; 2011040120 (ZJNU). *C. septempetala*; *S.S. Hong*; 2011040121 (ZJNU). *C. elongate*; *S.S. Hong*; 2011040122 (ZJNU). *C. campanisepala*; *S.S. Hong*; 2011040123 (ZJNU). *C. parvi-ovata*; *S.S. Hong*; 2011040124 (ZJNU). *C. lancicalyx*; *S.S. Hong*; 2011040125 (ZJNU). *C. tsofui*; *S.S. Hong*; 2011040126 (ZJNU). |
