# Supplementary material for: Classification of Camellia (Theaceae) Species Using Leaf Architecture Variations and Pattern Recognition Techniques
Source: PLoS One. 2012 Jan 3;7(1):e29704. doi: 10.1371/journal.pone.0029704 (PMC3250490; doi:10.1371/journal.pone.0029704)
Supplement: Table S1 — Species studied, as classified by Chang (1998). (DOC) [file pone.0029704.s002.doc]

**Table S1.** Species studied, as classified by Chang (1998)*.*

| **Sect. *Furfuracea*** ^1^ | **Sect. *Paracamellia*** ^2^ | **Sect. *Tuberculata*** ^3^ | **Sect. *Camellia*** ^4^ | **Sect. *Theopsis*** ^5^ |
| --- | --- | --- | --- | --- |
| Training set |  |  |  |  |
| 1. *C. pubifurfuracea* | 12. *C. grijsii* | 28. *C. tuberculata* | 40. *C. omeiensis* | 74. *C. macrosepala* |
| 2. *C. latipetiolata* | 13. *C.yuhsienensis* | 29. *C. rhytidocarpa* | 41. *C. polyodonta* | 75. *C. cuspidata var. grandiflora* |
| 3. *C. crapnalliana* | 14. *C. confusa* | 30. *C. anlungensis* | 42. *C. lanosituba* | 76. *C. forerrestii* |
| 4. *C. multibracteata* | 15. *C. kissi* | 31. *C. rubituberculata* | 43. *C. longigyna* | 77. *C. lipoensis* |
| 5. *C. furfuracea* | 16. *C. fluviatilis* | 32. *C. acuticalyx* | 44. *C. lapidea* | 78. *C. buxifolia* |
| 6. *C. oblate* | 17. *C. brevistyla* | 33. *C. atuberculata* | 45. *C. phelloderma* | 79. *C. minutiflora* |
|  | 18. *C. hiemalis* |  | 46. *C. mairei* | 80. *C. parvicuspidata* |
|  | 19. *C. obtusifolia* |  | 47. *C. villosa* | 81. *C. acutissima* |
|  |  |  | 48. *C. trichosperma* | 82. *C. handelii* |
|  |  |  | 49. *C. semiserrata* | 83. *C. costei* |
|  |  |  | 50. *C. reticulate* |  |
|  |  |  | 51. *C. semoserrata var. albiflora* |  |
|  |  |  | 52. *C. brevipetiolata* |  |
|  |  |  | 53. *C. phellocapsa* |  |
|  |  |  | 54. *C. compressa* |  |
|  |  |  | 55. *C. magniflora* |  |
|  |  |  | 56. *C. lungshenensis* |  |
|  |  |  | 57. *C. pitardii* |  |
| Test set |  |  |  |  |
| 7. *C. gaudichaudii* | 20. *C. maliflora* | 34. *C. obovatifolia* | 58. *C. pitardii var. Alba* | 84. *C. rosthorniana* |
| 8. *C. gigantocarpa* | 21. *C. shensiensis* | 35. *C. parvimuricata* | 59. *C.pitardii var. yunnaica* | 85. *C. euryoides* |
| 9. *C. octopetala* | 22. *C. puniceiflora* | 36. *C. Hupehensis* | 60. *C. oviformis* | 86. *C. parvilimba* |
| 10. *C. parfurfuracea* | 23. *C. tenii* | 37. *C. zengii* | 61. *C. saluenensis* | 87. *C. parvilimba var. brevipes* |

**Table S1. Continued**

| **Sect. *Furfuracea*** ^1^ | **Sect. *Paracamellia*** ^2^ | **Sect. *Tuberculata*** ^3^ | **Sect. *Camellia*** ^4^ | **Sect. *Theopsis*** ^5^ |
| --- | --- | --- | --- | --- |
| Test set |  |  |  |  |
| 11. *C. connatistyla* | 24. *C. miyagii* | 38. *C. pyxidiacea* | 62. *C. oligophlebia* | 88. *C. septempetala* |
|  | 25. *C. weiningensis* | 39. *C. crassifolia* | 63. *C. albo-sericea* | 89. *C. elongate* |
|  | 26. *C. odorata* |  | 64. *C. uraku* | 90. *C. campanisepala* |
|  | 27. *C. phaeoclada* |  | 65. *C. hilisciflora* | 91. *C. parvi-ovata* |
|  |  |  | 66. *C. delicate* | 92. *C. lancicalyx* |
|  |  |  | 67. *C. hunanica* | 93. *C. tsoful* |
|  |  |  | 68. *C. chekiangoleosa* |  |
|  |  |  | 69. *C. japonica* |  |
|  |  |  | 70. *C. rusticana* |  |
|  |  |  | 71. *C. crassissima* |  |
|  |  |  | 72. *C. apolyodonta* |  |
|  |  |  | 73. *C. longicaudata* |  |

^1, 2, 3, 4, 5^ represent the sample labels (categories) used in LVQ-ANN, DAN2 and SVM models.
