# Supplementary material for: Classification of Camellia (Theaceae) Species Using Leaf Architecture Variations and Pattern Recognition Techniques
Source: PLoS One. 2012 Jan 3;7(1):e29704. doi: 10.1371/journal.pone.0029704 (PMC3250490; doi:10.1371/journal.pone.0029704)
Supplement: Table S2 — Data matrix of characteristics on leaf architecture of genus Camellia. (DOC) [file pone.0029704.s003.doc]

**Table S2.** Data matrix of characteristics on leaf architecture of genus *Camellia* ^a^*.*

| Samples | Characteristics | | | | | | | | | | | | | | | | | | | | | | | | | | | | | | |
| --- | --- | --- | --- | --- | --- | --- | --- | --- | --- | --- | --- | --- | --- | --- | --- | --- | --- | --- | --- | --- | --- | --- | --- | --- | --- | --- | --- | --- | --- | --- | --- |
|  | 1 | 2 | 3 | 4 | 5 | 6 | 7 | 8 | 9 | 10 | 11 | 12 | 13 | 14 | 15 | 16 | 17 | 18 | 19 | 20 | 21 | 22 | 23 | 24 | 25 | 26 | 27 | 28 | 29 | 30 | 31 |
| 1 | 2 | 0 | 1 | 1 | 0 | 3 | 2 | 1 | 0 | 0 | 1 | 1 | 1 | 0 | 8 | 2 | 1 | 38.19 | 24.10 | 9.85 | 5.09 | 0.52 | 1.05 | 0.79 | 0.00 | 5.18 | 0.00 | 0.47 | 0.05 | 0.04 | 0.02 |
| 2 | 2 | 1 | 1 | 1 | 1 | 3 | 2 | 1 | 0 | 0 | 1 | 1 | 1 | 0 | 7 | 2 | 1 | 38.31 | 25.69 | 11.37 | 4.94 | 0.43 | 0.93 | 1.15 | 0.24 | 6.54 | 0.02 | 0.52 | 0.05 | 0.05 | 0.03 |
| 3 | 2 | 2 | 1 | 1 | 1 | 2 | 2 | 1 | 0 | 0 | 1 | 1 | 1 | 0 | 9 | 2 | 1 | 45.92 | 27.84 | 12.16 | 5.40 | 0.44 | 0.95 | 1.26 | 0.66 | 7.71 | 0.04 | 0.51 | 0.04 | 0.04 | 0.03 |
| 4 | 2 | 2 | 0 | 1 | 1 | 3 | 1 | 1 | 0 | 0 | 1 | 1 | 1 | 0 | 9 | 2 | 1 | 53.85 | 30.35 | 13.24 | 5.89 | 0.45 | 0.94 | 1.13 | 0.58 | 6.55 | 0.05 | 0.51 | 0.03 | 0.04 | 0.05 |
| 5 | 2 | 1 | 1 | 1 | 1 | 2 | 2 | 1 | 0 | 0 | 1 | 1 | 1 | 0 | 9 | 2 | 1 | 58.61 | 31.61 | 14.05 | 5.84 | 0.42 | 0.94 | 0.75 | 0.12 | 6.71 | 0.01 | 0.52 | 0.06 | 0.05 | 0.03 |
| 6 | 2 | 1 | 0 | 1 | 1 | 3 | 2 | 1 | 0 | 0 | 1 | 1 | 1 | 0 | 7 | 2 | 1 | 54.83 | 30.69 | 13.42 | 5.81 | 0.43 | 0.93 | 0.86 | 0.60 | 7.39 | 0.04 | 0.51 | 0.08 | 0.09 | 0.05 |
| 7 | 2 | 2 | 0 | 1 | 1 | 3 | 2 | 1 | 0 | 0 | 1 | 1 | 1 | 0 | 7 | 2 | 1 | 34.78 | 23.97 | 10.32 | 4.92 | 0.48 | 0.97 | 1.08 | 0.80 | 6.30 | 0.07 | 0.51 | 0.04 | 0.03 | 0.02 |
| 8 | 3 | 2 | 0 | 1 | 1 | 2 | 2 | 1 | 0 | 0 | 1 | 1 | 1 | 0 | 8 | 2 | 1 | 54.54 | 30.89 | 13.57 | 5.91 | 0.44 | 0.91 | 1.39 | 1.10 | 8.44 | 0.06 | 0.49 | 0.05 | 0.04 | 0.03 |
| 9 | 3 | 2 | 0 | 1 | 1 | 2 | 2 | 1 | 0 | 0 | 1 | 1 | 1 | 0 | 7 | 2 | 1 | 64.90 | 33.58 | 14.92 | 6.27 | 0.42 | 0.92 | 1.60 | 0.74 | 7.10 | 0.05 | 0.50 | 0.06 | 0.04 | 0.03 |
| 10 | 2 | 1 | 0 | 1 | 1 | 3 | 2 | 1 | 0 | 0 | 1 | 1 | 1 | 0 | 8 | 2 | 1 | 56.58 | 30.69 | 13.15 | 5.98 | 0.45 | 0.96 | 1.22 | 0.36 | 7.02 | 0.03 | 0.52 | 0.05 | 0.04 | 0.07 |
| 11 | 2 | 2 | 0 | 1 | 1 | 3 | 2 | 1 | 0 | 0 | 1 | 1 | 1 | 0 | 9 | 2 | 1 | 60.90 | 33.55 | 15.28 | 5.84 | 0.38 | 0.87 | 0.63 | 0.09 | 7.40 | 0.01 | 0.51 | 0.04 | 0.06 | 0.05 |
| 12 | 1 | 2 | 0 | 1 | 0 | 2 | 1 | 1 | 0 | 1 | 1 | 1 | 1 | 0 | 7 | 2 | 1 | 9.49 | 13.07 | 5.64 | 2.66 | 0.47 | 0.89 | 0.53 | 0.67 | 2.42 | 0.12 | 0.42 | 0.05 | 0.05 | 0.03 |
| 13 | 3 | 1 | 0 | 1 | 0 | 2 | 1 | 1 | 0 | 0 | 1 | 1 | 1 | 0 | 8 | 1 | 1 | 19.06 | 18.70 | 8.27 | 3.42 | 0.41 | 0.87 | 0.92 | 0.80 | 4.42 | 0.09 | 0.51 | 0.08 | 0.05 | 0.03 |
| 14 | 3 | 2 | 2 | 1 | 0 | 1 | 1 | 1 | 1 | 1 | 1 | 1 | 1 | 0 | 7 | 1 | 1 | 12.28 | 13.55 | 5.61 | 3.09 | 0.55 | 1.07 | 0.39 | 0.50 | 2.59 | 0.10 | 0.52 | 0.11 | 0.08 | 0.04 |
| 15 | 3 | 2 | 2 | 1 | 0 | 1 | 1 | 1 | 0 | 1 | 1 | 1 | 1 | 0 | 7 | 2 | 1 | 6.67 | 9.84 | 4.02 | 2.26 | 0.56 | 1.10 | 0.29 | 0.45 | 2.02 | 0.12 | 0.52 | 0.06 | 0.05 | 0.03 |
| 16 | 0 | 2 | 0 | 0 | 0 | 1 | 1 | 1 | 0 | 0 | 1 | 1 | 1 | 0 | 8 | 2 | 1 | 12.00 | 17.02 | 7.94 | 2.34 | 0.29 | 0.66 | 1.15 | 1.19 | 4.22 | 0.15 | 0.51 | 0.04 | 0.04 | 0.02 |
| 17 | 3 | 1 | 2 | 1 | 0 | 1 | 1 | 1 | 0 | 0 | 1 | 1 | 1 | 0 | 7 | 2 | 1 | 12.93 | 13.28 | 5.02 | 2.29 | 0.45 | 1.17 | 0.56 | 0.58 | 2.46 | 0.12 | 0.49 | 0.05 | 0.05 | 0.04 |
| 18 | 3 | 2 | 3 | 0 | 1 | 0 | 1 | 1 | 0 | 0 | 1 | 1 | 1 | 0 | 7 | 2 | 0 | 13.93 | 14.48 | 6.17 | 3.29 | 0.53 | 1.06 | 0.53 | 0.46 | 2.70 | 0.09 | 0.52 | 0.05 | 0.04 | 0.02 |
| 19 | 3 | 2 | 3 | 0 | 1 | 1 | 1 | 1 | 0 | 0 | 1 | 1 | 1 | 0 | 8 | 1 | 0 | 5.81 | 9.91 | 3.75 | 2.09 | 0.56 | 0.95 | 0.41 | 0.50 | 1.73 | 0.14 | 0.50 | 0.06 | 0.05 | 0.03 |
| 20 | 3 | 1 | 3 | 1 | 0 | 0 | 1 | 1 | 1 | 1 | 1 | 1 | 1 | 0 | 7 | 1 | 0 | 12.75 | 14.02 | 5.89 | 3.09 | 0.52 | 1.04 | 0.50 | 0.55 | 2.91 | 0.10 | 0.51 | 0.05 | 0.05 | 0.03 |
| 21 | 1 | 1 | 1 | 1 | 1 | 3 | 1 | 1 | 0 | 0 | 1 | 1 | 1 | 0 | 7 | 2 | 1 | 9.84 | 12.16 | 4.84 | 2.82 | 0.58 | 1.06 | 0.38 | 0.42 | 2.12 | 0.09 | 0.46 | 0.10 | 0.06 | 0.04 |
| 22 | 3 | 2 | 3 | 1 | 0 | 1 | 1 | 1 | 1 | 1 | 1 | 1 | 1 | 0 | 7 | 2 | 1 | 10.61 | 12.72 | 5.34 | 2.79 | 0.52 | 1.05 | 0.50 | 0.48 | 2.71 | 0.09 | 0.50 | 0.06 | 0.04 | 0.03 |

**Table S2.** Continued.

| Samples | Characteristics | | | | | | | | | | | | | | | | | | | | | | | | | | | | | | |
| --- | --- | --- | --- | --- | --- | --- | --- | --- | --- | --- | --- | --- | --- | --- | --- | --- | --- | --- | --- | --- | --- | --- | --- | --- | --- | --- | --- | --- | --- | --- | --- |
|  | 1 | 2 | 3 | 4 | 5 | 6 | 7 | 8 | 9 | 10 | 11 | 12 | 13 | 14 | 15 | 16 | 17 | 18 | 19 | 20 | 21 | 22 | 23 | 24 | 25 | 26 | 27 | 28 | 29 | 30 | 31 |
| 23 | 3 | 2 | 2 | 1 | 0 | 1 | 1 | 1 | 0 | 0 | 1 | 1 | 1 | 0 | 7 | 1 | 1 | 2.88 | 6.59 | 2.78 | 1.45 | 0.52 | 1.06 | 0.28 | 0.34 | 1.47 | 0.12 | 0.52 | 0.05 | 0.04 | 0.03 |
| 24 | 3 | 2 | 2 | 0 | 0 | 1 | 1 | 1 | 1 | 1 | 1 | 1 | 1 | 0 | 7 | 2 | 1 | 3.46 | 7.22 | 2.95 | 1.53 | 0.52 | 1.06 | 0.28 | 0.25 | 1.52 | 0.09 | 0.52 | 0.05 | 0.04 | 0.03 |
| 25 | 3 | 1 | 3 | 1 | 0 | 1 | 1 | 1 | 1 | 1 | 1 | 1 | 1 | 0 | 9 | 2 | 1 | 23.55 | 20.33 | 9.04 | 3.86 | 0.43 | 0.91 | 1.13 | 0.84 | 4.55 | 0.10 | 0.52 | 0.03 | 0.05 | 0.03 |
| 26 | 3 | 2 | 0 | 1 | 1 | 1 | 1 | 1 | 1 | 1 | 1 | 1 | 1 | 0 | 8 | 2 | 1 | 22.85 | 19.39 | 8.13 | 4.16 | 0.51 | 0.97 | 0.98 | 1.17 | 4.13 | 0.14 | 0.51 | 0.08 | 0.07 | 0.04 |
| 27 | 3 | 2 | 3 | 1 | 1 | 1 | 1 | 1 | 0 | 0 | 1 | 1 | 1 | 0 | 7 | 2 | 0 | 4.53 | 8.18 | 3.36 | 1.88 | 0.56 | 1.08 | 0.25 | 0.39 | 1.69 | 0.12 | 0.51 | 0.04 | 0.03 | 0.03 |
| 28 | 3 | 2 | 0 | 0 | 1 | 3 | 2 | 1 | 0 | 0 | 1 | 1 | 1 | 0 | 10 | 2 | 0 | 27.83 | 22.79 | 10.26 | 4.25 | 0.41 | 0.86 | 1.25 | 0.87 | 6.02 | 0.07 | 0.49 | 0.06 | 0.05 | 0.03 |
| 29 | 3 | 1 | 0 | 1 | 1 | 3 | 3 | 1 | 0 | 0 | 1 | 0 | 0 | 2 | 11 | 2 | 0 | 23.82 | 21.57 | 9.49 | 3.63 | 0.38 | 0.82 | 1.10 | 0.55 | 4.44 | 0.05 | 0.43 | 0.09 | 0.07 | 0.06 |
| 30 | 1 | 1 | 0 | 0 | 1 | 3 | 3 | 1 | 0 | 0 | 1 | 0 | 0 | 2 | 11 | 2 | 0 | 25.10 | 21.73 | 9.68 | 3.69 | 0.38 | 0.85 | 0.69 | 0.45 | 4.37 | 0.04 | 0.42 | 0.09 | 0.09 | 0.06 |
| 31 | 3 | 2 | 0 | 0 | 0 | 3 | 3 | 1 | 0 | 0 | 1 | 1 | 1 | 2 | 9 | 2 | 0 | 36.89 | 27.05 | 12.12 | 4.62 | 0.38 | 0.81 | 1.10 | 0.67 | 5.35 | 0.06 | 0.46 | 0.07 | 0.06 | 0.03 |
| 32 | 1 | 1 | 0 | 1 | 1 | 3 | 3 | 1 | 0 | 0 | 1 | 0 | 0 | 2 | 9 | 2 | 0 | 10.87 | 13.78 | 5.99 | 2.73 | 0.46 | 0.92 | 0.66 | 0.49 | 2.67 | 0.08 | 0.41 | 0.23 | 0.04 | 0.03 |
| 33 | 1 | 1 | 0 | 0 | 0 | 3 | 3 | 0 | 0 | 0 | 1 | 1 | 1 | 0 | 10 | 2 | 1 | 11.96 | 15.20 | 6.66 | 2.62 | 0.39 | 0.83 | 0.54 | 0.43 | 2.84 | 0.06 | 0.42 | 0.10 | 0.09 | 0.06 |
| 34 | 1 | 2 | 0 | 1 | 1 | 3 | 2 | 1 | 0 | 0 | 1 | 1 | 1 | 0 | 10 | 2 | 0 | 38.15 | 29.23 | 11.33 | 5.34 | 0.47 | 0.71 | 1.04 | 0.66 | 4.11 | 0.07 | 0.42 | 0.04 | 0.04 | 0.03 |
| 35 | 1 | 1 | 0 | 1 | 1 | 2 | 1 | 0 | 0 | 0 | 1 | 1 | 1 | 2 | 7 | 2 | 0 | 8.53 | 12.64 | 5.55 | 2.34 | 0.42 | 0.85 | 0.45 | 0.36 | 2.16 | 0.07 | 0.43 | 0.04 | 0.03 | 0.03 |
| 36 | 3 | 2 | 0 | 1 | 1 | 2 | 2 | 1 | 0 | 0 | 1 | 1 | 1 | 0 | 9 | 2 | 0 | 16.68 | 17.30 | 7.76 | 3.35 | 0.43 | 0.89 | 0.76 | 0.67 | 4.38 | 0.07 | 0.49 | 0.05 | 0.05 | 0.03 |
| 37 | 1 | 2 | 0 | 1 | 1 | 3 | 3 | 1 | 0 | 0 | 1 | 0 | 0 | 2 | 11 | 2 | 0 | 20.99 | 25.22 | 9.82 | 3.27 | 0.33 | 0.53 | 0.88 | 0.92 | 3.98 | 0.10 | 0.43 | 0.09 | 0.08 | 0.06 |
| 38 | 3 | 2 | 0 | 0 | 1 | 2 | 3 | 1 | 0 | 0 | 1 | 1 | 1 | 0 | 9 | 2 | 0 | 23.89 | 22.62 | 10.10 | 3.59 | 0.36 | 0.75 | 0.65 | 0.60 | 4.83 | 0.06 | 0.48 | 0.07 | 0.06 | 0.03 |
| 39 | 1 | 1 | 0 | 0 | 1 | 3 | 2 | 1 | 0 | 0 | 1 | 1 | 0 | 2 | 8 | 2 | 0 | 49.70 | 34.18 | 13.83 | 5.34 | 0.39 | 0.68 | 1.04 | 0.48 | 4.44 | 0.05 | 0.43 | 0.04 | 0.04 | 0.03 |
| 40 | 1 | 2 | 0 | 1 | 1 | 2 | 3 | 0 | 0 | 0 | 1 | 1 | 1 | 0 | 10 | 2 | 1 | 23.03 | 22.29 | 10.29 | 3.45 | 0.34 | 0.74 | 1.03 | 0.53 | 4.90 | 0.05 | 0.43 | 0.10 | 0.06 | 0.03 |
| 41 | 3 | 1 | 0 | 0 | 1 | 3 | 3 | 0 | 0 | 0 | 1 | 1 | 1 | 1 | 10 | 2 | 1 | 50.92 | 30.88 | 13.66 | 5.46 | 0.40 | 0.85 | 0.66 | 1.18 | 5.97 | 0.10 | 0.49 | 0.10 | 0.10 | 0.05 |
| 42 | 1 | 2 | 0 | 1 | 1 | 1 | 3 | 0 | 0 | 1 | 1 | 1 | 1 | 0 | 10 | 2 | 1 | 28.72 | 24.65 | 11.41 | 3.91 | 0.34 | 0.76 | 2.18 | 1.74 | 6.90 | 0.12 | 0.49 | 0.07 | 0.06 | 0.03 |
| 43 | 3 | 1 | 0 | 1 | 1 | 3 | 3 | 0 | 0 | 0 | 0 | 1 | 1 | 1 | 10 | 2 | 1 | 21.19 | 21.46 | 9.91 | 3.24 | 0.33 | 0.74 | 0.86 | 1.18 | 5.12 | 0.11 | 0.46 | 0.04 | 0.05 | 0.03 |
| 44 | 1 | 1 | 0 | 1 | 0 | 2 | 3 | 0 | 0 | 0 | 1 | 1 | 1 | 0 | 9 | 2 | 1 | 21.47 | 19.33 | 8.49 | 3.75 | 0.44 | 0.92 | 1.19 | 0.84 | 4.06 | 0.11 | 0.51 | 0.07 | 0.06 | 0.03 |
| 45 | 1 | 1 | 0 | 0 | 1 | 2 | 3 | 0 | 0 | 0 | 1 | 1 | 0 | 2 | 9 | 2 | 1 | 23.23 | 19.91 | 8.50 | 4.06 | 0.48 | 0.94 | 1.02 | 0.77 | 3.55 | 0.09 | 0.43 | 0.08 | 0.08 | 0.05 |

***Table S2.*** *Continued.*

| Samples | Characteristics | | | | | | | | | | | | | | | | | | | | | | | | | | | | | | |
| --- | --- | --- | --- | --- | --- | --- | --- | --- | --- | --- | --- | --- | --- | --- | --- | --- | --- | --- | --- | --- | --- | --- | --- | --- | --- | --- | --- | --- | --- | --- | --- |
|  | 1 | 2 | 3 | 4 | 5 | 6 | 7 | 8 | 9 | 10 | 11 | 12 | 13 | 14 | 15 | 16 | 17 | 18 | 19 | 20 | 21 | 22 | 23 | 24 | 25 | 26 | 27 | 28 | 29 | 30 | 31 |
| 46 | 1 | 1 | 0 | 1 | 1 | 3 | 3 | 0 | 0 | 0 | 1 | 1 | 1 | 0 | 10 | 2 | 1 | 28.27 | 23.02 | 10.05 | 4.24 | 0.42 | 0.85 | 0.76 | 1.28 | 3.95 | 0.14 | 0.43 | 0.07 | 0.08 | 0.04 |
| 47 | 1 | 1 | 0 | 1 | 1 | 3 | 3 | 0 | 0 | 0 | 1 | 1 | 0 | 2 | 10 | 2 | 1 | 22.22 | 20.30 | 8.71 | 3.90 | 0.45 | 0.86 | 0.79 | 0.82 | 3.94 | 0.09 | 0.43 | 0.09 | 0.07 | 0.03 |
| 48 | 3 | 1 | 0 | 0 | 1 | 2 | 3 | 1 | 0 | 0 | 1 | 1 | 1 | 0 | 7 | 2 | 1 | 43.06 | 24.94 | 10.22 | 5.72 | 0.56 | 1.11 | 1.53 | 6.40 | 4.90 | 0.63 | 0.49 | 0.07 | 0.11 | 0.00 |
| 49 | 1 | 1 | 0 | 1 | 1 | 1 | 3 | 1 | 0 | 1 | 1 | 1 | 0 | 2 | 9 | 2 | 1 | 69.08 | 34.25 | 14.87 | 6.91 | 0.46 | 0.94 | 1.49 | 6.19 | 6.25 | 0.49 | 0.49 | 0.09 | 0.10 | 0.00 |
| 50 | 1 | 1 | 0 | 1 | 1 | 2 | 3 | 1 | 0 | 0 | 1 | 1 | 1 | 0 | 10 | 2 | 1 | 16.36 | 17.68 | 7.94 | 3.23 | 0.41 | 0.84 | 0.81 | 1.02 | 3.99 | 0.12 | 0.48 | 0.04 | 0.04 | 0.03 |
| 51 | 1 | 1 | 0 | 0 | 1 | 1 | 2 | 1 | 0 | 0 | 1 | 1 | 1 | 0 | 10 | 2 | 1 | 49.41 | 30.30 | 13.27 | 5.55 | 0.42 | 0.86 | 1.32 | 2.45 | 6.06 | 0.17 | 0.43 | 0.04 | 0.03 | 0.02 |
| 52 | 1 | 1 | 0 | 1 | 1 | 2 | 3 | 0 | 0 | 0 | 1 | 1 | 1 | 0 | 9 | 2 | 1 | 44.49 | 27.11 | 11.08 | 6.03 | 0.54 | 0.97 | 1.72 | 1.10 | 5.13 | 0.10 | 0.47 | 0.12 | 0.10 | 0.04 |
| 53 | 1 | 1 | 0 | 1 | 1 | 2 | 3 | 0 | 0 | 0 | 1 | 1 | 0 | 2 | 9 | 2 | 1 | 27.50 | 23.05 | 10.29 | 3.94 | 0.38 | 0.83 | 0.56 | 0.42 | 4.17 | 0.05 | 0.44 | 0.03 | 0.02 | 0.01 |
| 54 | 3 | 2 | 0 | 1 | 1 | 1 | 3 | 0 | 0 | 0 | 1 | 1 | 1 | 0 | 11 | 2 | 1 | 42.90 | 30.78 | 14.18 | 4.43 | 0.31 | 0.72 | 1.92 | 0.83 | 7.59 | 0.06 | 0.51 | 0.06 | 0.05 | 0.03 |
| 55 | 3 | 2 | 0 | 1 | 1 | 1 | 3 | 0 | 0 | 0 | 1 | 1 | 1 | 0 | 10 | 2 | 1 | 42.43 | 27.29 | 12.00 | 5.21 | 0.43 | 0.91 | 1.19 | 1.41 | 6.18 | 0.12 | 0.51 | 0.08 | 0.06 | 0.03 |
| 56 | 3 | 2 | 0 | 1 | 1 | 2 | 3 | 1 | 0 | 0 | 0 | 1 | 1 | 1 | 10 | 2 | 1 | 22.13 | 21.25 | 9.64 | 3.70 | 0.38 | 0.78 | 1.01 | 3.57 | 6.12 | 0.29 | 0.49 | 0.07 | 0.05 | 0.02 |
| 57 | 1 | 2 | 0 | 1 | 1 | 1 | 3 | 1 | 0 | 0 | 1 | 1 | 1 | 0 | 9 | 2 | 1 | 19.02 | 19.51 | 8.82 | 3.36 | 0.38 | 0.80 | 1.00 | 0.88 | 3.85 | 0.10 | 0.44 | 0.07 | 0.04 | 0.02 |
| 58 | 3 | 2 | 0 | 0 | 1 | 1 | 3 | 1 | 0 | 0 | 1 | 1 | 1 | 0 | 9 | 2 | 1 | 11.22 | 14.48 | 6.42 | 2.60 | 0.40 | 0.86 | 0.87 | 0.32 | 3.23 | 0.05 | 0.50 | 0.07 | 0.05 | 0.03 |
| 59 | 1 | 1 | 0 | 0 | 0 | 1 | 3 | 0 | 0 | 0 | 1 | 1 | 1 | 0 | 10 | 2 | 1 | 31.91 | 24.41 | 10.63 | 4.66 | 0.44 | 0.86 | 1.12 | 0.26 | 4.31 | 0.02 | 0.40 | 0.13 | 0.09 | 0.09 |
| 60 | 3 | 1 | 0 | 1 | 1 | 2 | 2 | 1 | 1 | 1 | 1 | 1 | 1 | 0 | 11 | 2 | 1 | 32.70 | 22.80 | 9.76 | 4.96 | 0.51 | 1.01 | 1.02 | 0.63 | 5.28 | 0.06 | 0.47 | 0.05 | 0.03 | 0.02 |
| 61 | 3 | 2 | 0 | 0 | 1 | 1 | 3 | 0 | 0 | 0 | 1 | 1 | 1 | 0 | 9 | 2 | 1 | 9.79 | 13.58 | 6.11 | 2.50 | 0.41 | 0.85 | 0.68 | 0.77 | 3.27 | 0.11 | 0.48 | 0.31 | 0.05 | 0.03 |
| 62 | 1 | 2 | 0 | 1 | 1 | 2 | 3 | 1 | 0 | 0 | 1 | 1 | 1 | 0 | 7 | 2 | 1 | 9.83 | 13.02 | 5.69 | 2.46 | 0.43 | 0.93 | 1.30 | 0.41 | 4.27 | 0.04 | 0.43 | 0.07 | 0.05 | 0.04 |
| 63 | 1 | 1 | 0 | 1 | 1 | 3 | 3 | 1 | 0 | 0 | 1 | 1 | 1 | 0 | 11 | 2 | 1 | 27.99 | 21.91 | 9.53 | 4.26 | 0.45 | 0.93 | 0.52 | 0.21 | 4.45 | 0.02 | 0.45 | 0.06 | 0.05 | 0.02 |
| 64 | 1 | 1 | 0 | 0 | 1 | 1 | 2 | 1 | 0 | 0 | 1 | 1 | 1 | 0 | 9 | 2 | 1 | 25.84 | 21.19 | 9.27 | 4.15 | 0.45 | 0.92 | 1.00 | 0.61 | 4.37 | 0.07 | 0.48 | 0.06 | 0.04 | 0.02 |
| 65 | 3 | 2 | 0 | 0 | 1 | 2 | 3 | 1 | 0 | 0 | 1 | 1 | 1 | 0 | 10 | 2 | 1 | 19.34 | 19.24 | 8.77 | 3.33 | 0.38 | 0.84 | 1.24 | 1.11 | 4.23 | 0.13 | 0.50 | 0.06 | 0.06 | 0.05 |
| 66 | 1 | 1 | 0 | 0 | 1 | 2 | 3 | 1 | 0 | 1 | 1 | 1 | 0 | 2 | 10 | 2 | 1 | 21.27 | 18.26 | 7.67 | 3.92 | 0.51 | 1.02 | 0.97 | 0.24 | 3.07 | 0.03 | 0.42 | 0.05 | 0.04 | 0.02 |
| 67 | 1 | 1 | 0 | 1 | 1 | 1 | 3 | 1 | 0 | 0 | 1 | 1 | 1 | 0 | 8 | 2 | 1 | 25.32 | 20.80 | 8.68 | 4.35 | 0.50 | 0.94 | 0.48 | 0.42 | 3.52 | 0.05 | 0.42 | 0.07 | 0.05 | 0.03 |
| 68 | 4 | 2 | 0 | 1 | 1 | 1 | 1 | 1 | 1 | 0 | 1 | 1 | 1 | 0 | 9 | 2 | 1 | 21.58 | 18.85 | 7.99 | 4.76 | 0.60 | 0.97 | 1.06 | 1.20 | 4.53 | 0.14 | 0.51 | 0.04 | 0.04 | 0.03 |

**Table S2.** Continued.

| Samples | Characteristics | | | | | | | | | | | | | | | | | | | | | | | | | | | | | | |
| --- | --- | --- | --- | --- | --- | --- | --- | --- | --- | --- | --- | --- | --- | --- | --- | --- | --- | --- | --- | --- | --- | --- | --- | --- | --- | --- | --- | --- | --- | --- | --- |
|  | 1 | 2 | 3 | 4 | 5 | 6 | 7 | 8 | 9 | 10 | 11 | 12 | 13 | 14 | 15 | 16 | 17 | 18 | 19 | 20 | 21 | 22 | 23 | 24 | 25 | 26 | 27 | 28 | 29 | 30 | 31 |
| 69 | 4 | 2 | 0 | 1 | 1 | 1 | 1 | 1 | 0 | 0 | 1 | 1 | 1 | 0 | 9 | 2 | 1 | 15.04 | 15.05 | 6.16 | 3.77 | 0.61 | 1.06 | 1.00 | 1.01 | 4.37 | 0.12 | 0.52 | 0.06 | 0.04 | 0.02 |
| 70 | 3 | 2 | 0 | 1 | 1 | 2 | 2 | 1 | 0 | 0 | 1 | 1 | 1 | 0 | 8 | 1 | 1 | 23.92 | 20.43 | 9.05 | 3.86 | 0.43 | 0.92 | 0.66 | 1.24 | 4.79 | 0.13 | 0.52 | 0.03 | 0.03 | 0.01 |
| 71 | 3 | 2 | 0 | 1 | 1 | 2 | 3 | 1 | 0 | 0 | 1 | 1 | 1 | 0 | 10 | 1 | 1 | 53.50 | 30.86 | 13.45 | 6.04 | 0.45 | 0.90 | 1.78 | 4.05 | 6.74 | 0.29 | 0.48 | 0.17 | 0.14 | 0.07 |
| 72 | 3 | 1 | 0 | 0 | 1 | 3 | 3 | 0 | 0 | 0 | 1 | 1 | 1 | 0 | 11 | 2 | 1 | 27.57 | 22.34 | 9.84 | 4.15 | 0.42 | 0.88 | 0.83 | 0.62 | 5.52 | 0.05 | 0.48 | 0.06 | 0.07 | 0.04 |
| 73 | 3 | 2 | 0 | 1 | 1 | 1 | 3 | 1 | 0 | 0 | 1 | 1 | 1 | 0 | 9 | 2 | 1 | 19.93 | 19.71 | 8.75 | 3.42 | 0.39 | 0.82 | 1.33 | 0.33 | 4.74 | 0.04 | 0.51 | 0.08 | 0.04 | 0.02 |
| 74 | 1 | 0 | 0 | 0 | 0 | 1 | 1 | 0 | 0 | 0 | 1 | 1 | 1 | 0 | 9 | 2 | 1 | 14.56 | 15.94 | 6.49 | 3.25 | 0.50 | 0.92 | 0.39 | 0.16 | 1.97 | 0.03 | 0.38 | 0.04 | 0.04 | 0.02 |
| 75 | 1 | 1 | 0 | 1 | 1 | 0 | 1 | 0 | 0 | 1 | 1 | 1 | 1 | 0 | 9 | 2 | 1 | 14.19 | 16.39 | 6.69 | 3.35 | 0.50 | 0.85 | 0.40 | 0.87 | 2.58 | 0.14 | 0.40 | 0.03 | 0.04 | 0.02 |
| 76 | 1 | 1 | 0 | 1 | 0 | 0 | 1 | 0 | 1 | 0 | 1 | 1 | 1 | 1 | 8 | 2 | 1 | 2.56 | 7.45 | 3.44 | 1.18 | 0.34 | 0.74 | 0.15 | 0.58 | 1.50 | 0.18 | 0.46 | 0.03 | 0.03 | 0.02 |
| 77 | 4 | 1 | 0 | 0 | 0 | 0 | 1 | 0 | 0 | 0 | 1 | 1 | 1 | 0 | 6 | 2 | 1 | 3.49 | 8.13 | 3.18 | 1.83 | 0.58 | 0.84 | 0.15 | 0.59 | 1.37 | 0.19 | 0.44 | 0.03 | 0.03 | 0.02 |
| 78 | 3 | 1 | 0 | 1 | 0 | 0 | 1 | 0 | 1 | 0 | 1 | 1 | 1 | 0 | 8 | 2 | 1 | 2.73 | 7.45 | 3.41 | 1.23 | 0.36 | 0.79 | 0.29 | 0.82 | 2.17 | 0.19 | 0.50 | 0.03 | 0.03 | 0.01 |
| 79 | 1 | 1 | 0 | 1 | 0 | 0 | 1 | 0 | 0 | 0 | 1 | 1 | 0 | 0 | 12 | 1 | 1 | 4.41 | 10.63 | 4.95 | 1.49 | 0.30 | 0.62 | 0.48 | 0.95 | 1.73 | 0.23 | 0.42 | 0.03 | 0.03 | 0.01 |
| 80 | 1 | 1 | 0 | 0 | 0 | 0 | 1 | 0 | 0 | 0 | 1 | 1 | 1 | 0 | 11 | 2 | 1 | 3.86 | 8.95 | 4.16 | 1.52 | 0.37 | 0.77 | 0.18 | 0.41 | 1.53 | 0.11 | 0.40 | 0.10 | 0.02 | 0.01 |
| 81 | 1 | 2 | 0 | 0 | 0 | 0 | 1 | 0 | 0 | 0 | 1 | 1 | 1 | 0 | 8 | 1 | 1 | 8.02 | 12.38 | 5.50 | 2.33 | 0.42 | 0.84 | 0.26 | 0.75 | 1.82 | 0.19 | 0.46 | 0.02 | 0.02 | 0.02 |
| 82 | 1 | 1 | 0 | 1 | 0 | 0 | 1 | 0 | 0 | 1 | 1 | 1 | 1 | 1 | 11 | 1 | 1 | 6.25 | 10.76 | 4.77 | 2.05 | 0.43 | 0.86 | 0.38 | 0.69 | 2.21 | 0.14 | 0.45 | 0.03 | 0.03 | 0.02 |
| 83 | 3 | 2 | 0 | 1 | 0 | 0 | 1 | 0 | 1 | 0 | 1 | 1 | 1 | 0 | 7 | 2 | 1 | 4.32 | 9.00 | 4.02 | 1.71 | 0.42 | 0.85 | 0.43 | 0.44 | 2.51 | 0.09 | 0.50 | 0.04 | 0.04 | 0.02 |
| 84 | 1 | 1 | 0 | 1 | 0 | 0 | 1 | 0 | 0 | 0 | 1 | 1 | 1 | 0 | 11 | 2 | 1 | 7.94 | 13.04 | 5.25 | 2.39 | 0.46 | 0.75 | 0.39 | 0.53 | 2.38 | 0.09 | 0.41 | 0.02 | 0.06 | 0.05 |
| 85 | 1 | 1 | 0 | 1 | 0 | 0 | 0 | 0 | 0 | 0 | 1 | 1 | 1 | 0 | 9 | 2 | 1 | 3.77 | 8.30 | 3.58 | 1.59 | 0.44 | 0.88 | 0.34 | 4.51 | 1.82 | 1.00 | 0.40 | 0.00 | 0.00 | 0.00 |
| 86 | 3 | 2 | 0 | 1 | 1 | 0 | 1 | 0 | 0 | 0 | 1 | 1 | 1 | 0 | 7 | 2 | 1 | 2.62 | 7.54 | 3.15 | 1.32 | 0.42 | 0.74 | 0.34 | 0.71 | 1.98 | 0.18 | 0.51 | 0.03 | 0.03 | 0.03 |
| 87 | 3 | 2 | 0 | 1 | 0 | 0 | 1 | 0 | 0 | 0 | 1 | 1 | 1 | 0 | 7 | 2 | 1 | 4.46 | 8.75 | 3.77 | 1.87 | 0.50 | 0.93 | 0.18 | 1.08 | 1.89 | 0.29 | 0.51 | 0.03 | 0.03 | 0.01 |
| 88 | 1 | 1 | 0 | 1 | 0 | 0 | 1 | 0 | 1 | 0 | 0 | 0 | 0 | 0 | 9 | 1 | 1 | 8.22 | 11.82 | 5.08 | 2.42 | 0.48 | 0.94 | 0.21 | 0.62 | 1.68 | 0.17 | 0.46 | 0.01 | 0.01 | 0.01 |
| 89 | 1 | 1 | 0 | 1 | 1 | 0 | 1 | 0 | 0 | 0 | 1 | 1 | 1 | 0 | 10 | 2 | 1 | 5.18 | 9.26 | 4.34 | 1.21 | 0.28 | 0.97 | 0.38 | 0.52 | 1.70 | 0.12 | 0.38 | 0.02 | 0.03 | 0.02 |
| 90 | 1 | 1 | 0 | 0 | 0 | 0 | 1 | 0 | 0 | 1 | 1 | 1 | 1 | 0 | 11 | 1 | 1 | 9.43 | 12.42 | 5.16 | 2.82 | 0.55 | 0.98 | 0.39 | 0.89 | 2.17 | 0.18 | 0.43 | 0.02 | 0.02 | 0.02 |
| 91 | 1 | 2 | 0 | 0 | 0 | 0 | 1 | 0 | 1 | 1 | 0 | 1 | 1 | 1 | 7 | 2 | 1 | 4.16 | 9.75 | 4.36 | 1.66 | 0.38 | 0.70 | 0.13 | 0.79 | 1.78 | 0.18 | 0.40 | 0.02 | 0.05 | 0.03 |

**Table S2.** Continued.

| Samples | Characteristics | | | | | | | | | | | | | | | | | | | | | | | | | | | | | | |
| --- | --- | --- | --- | --- | --- | --- | --- | --- | --- | --- | --- | --- | --- | --- | --- | --- | --- | --- | --- | --- | --- | --- | --- | --- | --- | --- | --- | --- | --- | --- | --- |
|  | 1 | 2 | 3 | 4 | 5 | 6 | 7 | 8 | 9 | 10 | 11 | 12 | 13 | 14 | 15 | 16 | 17 | 18 | 19 | 20 | 21 | 22 | 23 | 24 | 25 | 26 | 27 | 28 | 29 | 30 | 31 |
| 92 | 1 | 2 | 0 | 0 | 0 | 0 | 1 | 0 | 0 | 1 | 1 | 1 | 1 | 0 | 13 | 2 | 1 | 7.05 | 12.10 | 5.48 | 2.01 | 0.37 | 0.77 | 0.37 | 0.47 | 2.32 | 0.09 | 0.43 | 0.03 | 0.03 | 0.02 |
| 93 | 1 | 1 | 0 | 0 | 0 | 0 | 1 | 0 | 1 | 1 | 1 | 1 | 0 | 2 | 7 | 2 | 1 | 12.71 | 14.44 | 5.98 | 3.32 | 0.55 | 0.98 | 0.41 | 0.86 | 2.46 | 0.15 | 0.43 | 0.05 | 0.06 | 0.03 |

^a^ The number in samples’ column means the material mutual correspondence to Table 1. The number in characteristics’ row means the leaf architecture trait tested. Number 1-31 represent whole lamina shape (1); base only (2); apex (3); abaxial surface (4); adaxial surface (5); reticulate veins (6); margin shape (7); margin spacing (8); secondary veins shape (9); secondary veins balance (10); angulation between secondary veins and primary veins on upper part (11), on middle part (12), and on lower part (13); variations in angle of divergence of secondary veins (14); number of secondary veins (pairs) (15); veinlets (16); areoles development (17); leaf area (cm^2^) (18); perimeter (cm) (19); vertical length (cm) (20); horizontal width (cm) (21); leaf aspect ration (22); leaf form factor (23), petiole length (cm) (24); average value of entirely vein height (EVH) (cm) (25); average value of leaf widest part height (LWPH) (cm) (26); ratio of EVH and leaf vertical length (27); ratio of LWPH and leaf vertical length (28); serrulate length in upper part of leaf (29); serrulate length in middle part of leaf (30); and serrulate length in lower part of leaf (cm) (31), respectively.
